# Blaunet: An R-based graphical user interface package to analyze Blau space

**DOI:** 10.1371/journal.pone.0204990

**Published:** 2018-10-01

**Authors:** Michael Genkin, Cheng Wang, George Berry, Matthew E. Brashears

**Affiliations:** 1 School of Social Sciences, Singapore Management University, Singapore, Singapore; 2 Department of Sociology, University of Notre Dame, Notre Dame, IN, United States of America; 3 Department of Sociology, Cornell University, Ithaca, NY, United States of America; 4 Department of Sociology, University of South Carolina, Columbia, SC, United States of America; University of Michigan, UNITED STATES

## Abstract

McPherson’s Blau space and affiliation ecology model is a powerful tool for analyzing the ecological competition among social entities, such as organizations, along a combination of sociodemographic characteristics of their members. In this paper we introduce the R-based Graphical User Interface (GUI) package Blaunet, an integrated set of tools to calculate, visualize, and analyze the statuses of individuals and social entities in Blau space, parameterized by multiple sociodemographic traits as dimensions. The package is able to calculate the Blau statuses at the nodal, dyadic, and meso levels based on three types of information: sociodemographic characteristics, group affiliations (e.g., membership in groups/organizations), and network ties. To facilitate this, Blaunet has the following five main capabilities, it can: 1) identify a list of possible salient dimensions; 2) calculate, plot, and analyze niches for social entities by measuring the social distance along the salient dimensions between individuals affiliated with them; 3) generate Blau bubbles for individuals, thereby allowing the study of interpersonal influence of similar others even with limited or no network information; 4) capture niche dynamics cross-sectionally by calculating the intensity of exploitation from the carrying capacity and the membership rate; and 5) analyze the niche movement longitudinally by estimating the predicted niche movement equations. We illustrate these capabilities of Blaunet with example datasets.

## Introduction

Blaunet is based on two fundamental concepts in sociology: Blau space and affiliation ecology. Blau space rests on the idea that human beings can be represented in a *k*-dimensional space, where continuous sociodemographic characteristics such as age, income, and years of education serve as the dimensions. The dimensions that structure Blau space are socially salient (i.e., they influence association probabilities) and are referred to as Blau parameters [1, 2] in honor of the eminent sociologist Peter Blau. Each person’s attributes on the Blau parameters define his or her location in the Blau space, which in turn results in the individual’s affiliation to social entities such as organizations, cultural tastes, and political preferences, while those entities are more or less competing with one another in this space.

The R-based package Blaunet, accessible via the Graphical User Interface (GUI), is built for Blau space and affiliation ecology models. In particular, Blaunet contains an integrated set of tools for accomplishing five important and interrelated tasks: salient dimension identification, niche plotting, Blau bubble (proximity) analysis, niche analysis, and niche dynamics analysis. Many of these tools provide an easy way to calculate and visualize some of the widely used techniques necessary for analyzing Blau space. The identification of salient dimensions is an especially useful tool as it offers a principled way of selecting the dimensions that constitute Blau space and that underpin this kind of analysis. This paper focuses on an overview and description of the functionality of the Blaunet package. The remaining paper proceeds in four sections. Below we elaborate the theoretical framework underlying Blaunet in greater depth. The “Materials and Methods” section describes the installation instructions and the example datasets included in the Blaunet package. The “Results” section illustrates some of Blaunet’s major capabilities. And the “Discussion” section concludes with a discussion of some of Blaunet’s present limitations as well as some areas for future development. We believe this package will be useful to scholars from a variety of backgrounds as Blau space ideas have diffused from sociology into a diverse array of disciplines, including demography [3], criminology [4, 5], geography [6], psychology [7], political science [8, 9], business schools [10], and regional science [11]. Similarly, the Blaunet package should be useful to computer scientists, engineers, and applied scientists who study networks or incorporate sociological insights such as Blau space into their work [12–16]. In particular, those interested in the study of organizations and culture, social capital, and affiliation researchers, as well as those who wish to incorporate social space into their models or infer network effects from limited or absent network information may find Blau space approaches and the Blaunet package of value.

### Theoretical framework

The Blaunet package is based on the Blau space and affiliation ecology model, which uses the homophily principle to construct ecological models of a variety of social entities.

Homophily, or the tendency for individuals to associate with those like themselves, has long been considered a key element of social structure [17, 18]. Two types of homophily are categorized in the foundational work of Lazarsfeld and Merton–status homophily which may be premised on either ascribed (fixed) characteristics of the person (e.g., ethnoracial status, gender, birth date, etc.) or acquired (changeable) social markers (e.g., religion, education, occupation, etc.), and value homophily related to similarities in attitudes, values, practices, and beliefs [17]. More recent work has shifted focus to distinguish between structural homophily (i.e., tendency to be sorted into contexts containing similar others) and choice homophily (i.e., tendency to select similar others from among those available) [18]. Either way, however, there is broad agreement that homophily is a critical driver of association.

Blau’s macrostructural theory [19–21] extended the theory of homophily by arguing that macro-level social structure is fundamentally comprised of micro-level social positions which are parameterized by a family of sociodemographic factors that segregate homogenous people into the same areas in latent social space. Likewise, those who are non-identical, but similar, are relatively proximate in this space, with increasing dissimilarity producing decreasing proximity. Whether two individuals interact with each other depends on how they are distributed in this latent social space. Those positioned in the same areas of the latent social space are more likely to occupy the same foci (e.g., workplace, neighborhood, etc.) [22] and thus are more likely to come into contact with one another [19–21].

Miller McPherson named the above-mentioned latent social space “Blau space” in honor of “Peter Blau’s fundamental contribution to our understanding of system-level consequences of the organization of the sociodemographic variables” [2]. Blau space is therefore a *k*-dimensional hyperspace coordinate system, created by considering the set of *k* sociodemographic variables as dimensions or axes [1, 2, 23].

In Blau space, individuals occupy positions determined by their particular combinations of sociodemographic traits. Note that not all possible locations are occupied by actual individuals and there can be vast empty areas in which particular configurations of sociodemographic characteristics are not found together (e.g., there are relatively few ten year olds who have a college degree.) Based on the homophily principle, individuals are more likely to form social networks with others close to each other in Blau space. Because most recruitment to groups/organizations occur through the social relations of their existing members [24], and individual networks are constrained by homophily [18], organizations tend to recruit from localized areas of Blau space. These areas are termed “niches” and it is through them that social entities, such as organizations, are localized to particular areas of Blau space. Thus the Blau space and affiliation ecology model provides a theoretical skeleton around which individuals (e.g., members), social networks (e.g., social relationships among individuals), and the social entities (e.g., groups/organizations) are linked in a common multi-level framework.

McPherson [1] argues that organizations are social entities competing for their members’ time and energy. Because the total amount of human time and energy available in an area of Blau space is limited, the pressures of variation, selection, and retention apply to social entities competing in this space. Blau space therefore comprises an ecological model of social entities. Perhaps not surprisingly, the approach draws heavily on ideas from biological ecology, and can thus be applied in many similar ways. For example, if two organizations have overlapping niches, they are drawing from the same pool of potential members, and will compete with each other not unlike how two bacterial colonies compete for nutrients. If there are too many organizations situated in a certain area of Blau space (e.g., where the supply of potential members is substantially below the demands of the recruiting organizations), organizational niches will shift towards the relatively low competition areas. In such areas, the supply of potential members exceeds the demands of the recruiting organizations, thereby providing an opportunity for organizations to recruit members while avoiding competitive pressure. McPherson and Ranger-Moore [23] used this specific idea to predict niche movements of 16 types of voluntary organizations over 9 years, finding an impressive concordance between prediction and observation. This perspective, formally restated in mathematical terms by Péli & Bruggeman [25] and evaluated further by McPherson, Popielarz, and Drobnic [26] and Hannan, Pólos, and Carroll [27], has received significant support and is a productive way to explain change in the mean of the niche, the change in the dispersion of the niche, as well as the change in the density of exploitation of the niche. In this way the Blau space and affiliation ecology model has been shown to accurately predict aspects of various types of groups and organizations [1, 23, 28–32]. Moreover, it has been broadened to include cultural constructs such as preferences, identities, cultural artifacts, meanings, and institutional logics [33–37] and applied to the functional roles of cities [38]. Lastly the Blau space and affiliation ecology model has also been applied to the development of individual behavior [39–41], the formation and evolution of social networks [42, 43] and communities [13], the adoption and diffusion of innovations [15], and even civil war [9].

The Blau space and affiliation ecology model is advantageous in that it is applicable to almost any social survey variable. It provides a metric for understanding the relationships among a wide variety of social entities, and is a dynamic model which combines entities into populations, communities, and (both organizational and cultural) systems. It is also a powerful tool for understanding how conventional relational networks and group affiliation dynamics interact with one another [44], even when network data are either limited or unavailable.

## Materials and methods

### Blaunet installation

Blaunet is an open-source R-based package available at https://cran.r-project.org/web/packages/Blaunet/index.html. Users are recommended to have the most recent version of R installed. Blaunet can be installed and used on Windows, Linux, and OS X machines. Once installed it behaves very similarly regardless of platform, but the installation steps vary.

For Microsoft Windows users, Blaunet can be installed directly in R via

**R> install.packages('Blaunet', repos = "http://cran.r-project.org", dependencies = TRUE)**

Linux users should have X11 installed and GTK+ environment built in terminal via

**$ sudo apt-get install libglpk-dev**

**$ sudo apt-get install libgtk2.0-dev**

**$ sudo apt-get build-dep r-cran-rgl**

Then install Blaunet in R via

**R> install.packages('Blaunet', repos = "http://cran.r-project.org", dependencies = TRUE)**

The installation procedures are more involved for OS X users. More details are provided in the S1 File *Manual for the Blaunet Graphic User Interface Package*.

### Loading Blaunet

Blaunet can be loaded in R via

**R> library(Blaunet)**

**R> blaunetgui()**

**Fig 1:**
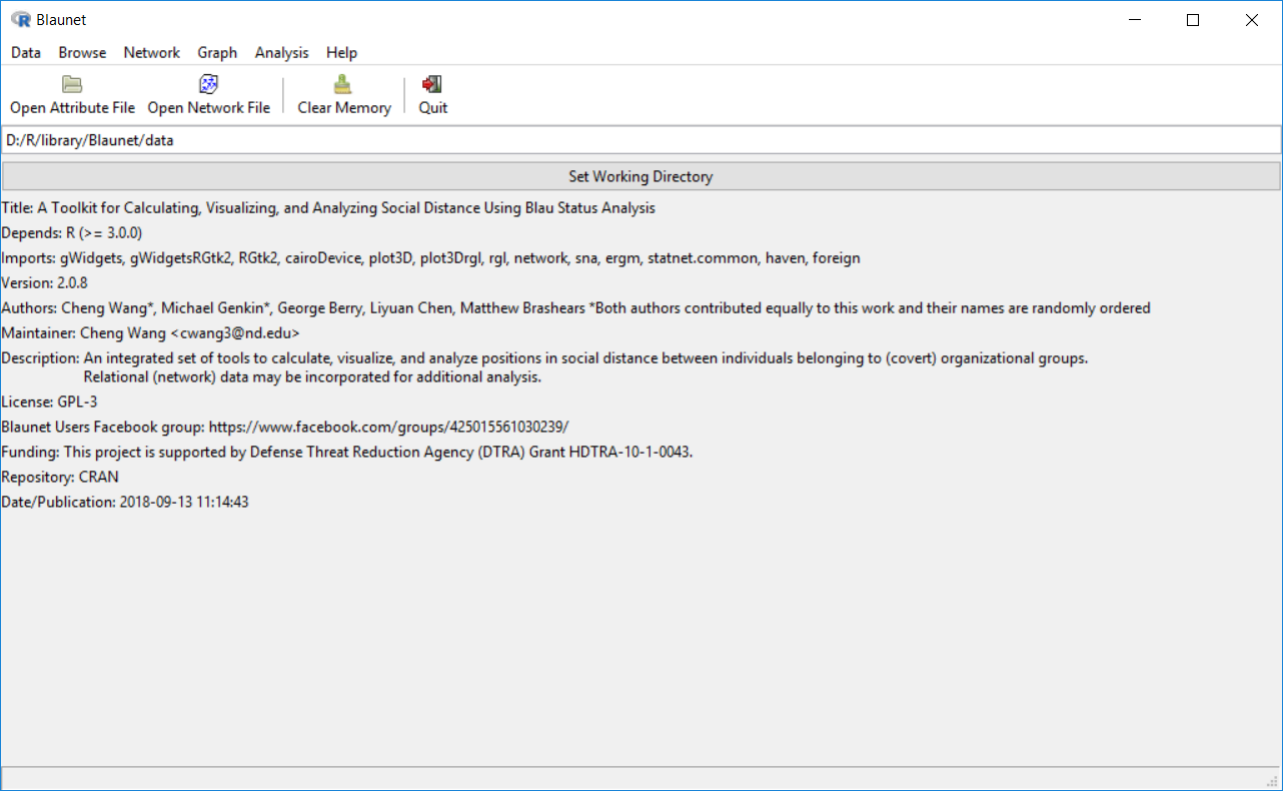
Main interface of Blaunet.

A first-time installation of Blaunet will install a number of dependent packages (see section below). Blaunet will be launched in a new window as a menu-based graphical user interface, in the manner of Fig 1. As shown in Fig 1, the main interface of Blaunet includes five elements–the menu bar, the toolbar, the text frame showing current working directory, the “Set Working Directory” bar, and the general information of Blaunet, from top to bottom.

### Dependent packages

It may take some time for Blaunet to be loaded for the first time no matter what type of operating system one’s computer is running, because several dependent packages are down-loaded and installed automatically, including:

(1–4) gWidgets, gWidgetsRGtk2, RGtk2, and cairoDevice packages, which function to build the graphic interface of Blaunet;

(5–7) plot3D, plot3Drgl, and rgl packages, which function to make 3D plots;

(8–11) network, sna, ergm, and statnet.common packages, which function to do network operations and analysis; and

(12–13) haven and foreign packages, which function to import and export data file between R and other statistical software, such as SAS, SPSS, Stata, and Microsoft Excel.

Upon executing blaunetgui() for the first time, Microsoft Windows users will be prompted to install GTK++ if they do not have it installed already

### Example datasets in Blaunet

Blaunet handles three types of information–sociodemographic information that can be used as Blau parameters, group affiliation information that can be used to build the niche for each social entity in the Blau space, and network information that indicates the relationships among the individuals–separately or jointly.

Blaunet users can utilize either the first two items under the “Data” menu or the first two icons on the toolbar to load the attribute and network files. *Caution*: one should be careful to not load the network data as attribute data or the attribute data as network data. It does not matter which one is loaded first. Before opening a new dataset, the user should clear the memory so that the data of the next project will not be corrupted by the data of the previous project. This can be done by clicking the “Clear Memory” item from the “Data” menu or the third icon from the toolbar.

**Fig 2:**
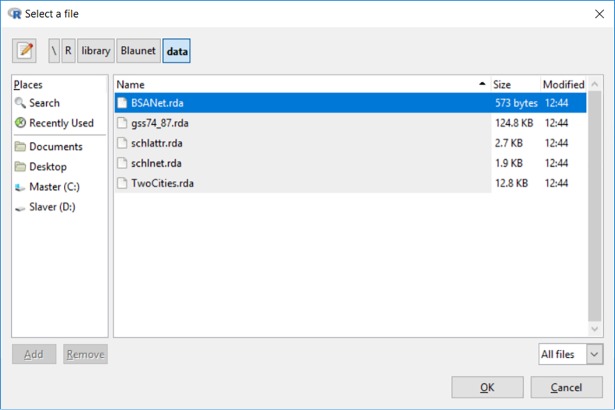
Interface for loading data.

The Blaunet package provides four example datasets, two of which contain all three types of information (e.g., *BSANet*.*rda*, and *schlattr*.*rda* & *schlnet*.*rda*) while the other two contain the sociodemographic and group affiliation information, but no network information (*TwoCities*.*rda* and *gss74_87*.*rda*). *BSANet*.*rda* is an artificial dataset containing no information of any real person, and the other 3 example datasets (*schlattr*.*rda* & *schlnet*.*rda*, *TwoCities*.*rda*, and *gss74_87*.*rda*) are fully anonymized and de-identified. As shown in Fig 2, all the example datasets are available under the “data” folder, a subdirectory of the Blaunet install location.

*BSANet*.*rda*. This is a small dataset containing 10 individuals in two non-overlapping locations (New York and San Francisco), created solely to illustrate the functions of the Blaunet package. It contains sociodemographic information such as the individuals’ age, education, and income; group affiliation information of memberships in a liberal or conservative organization (or both); and network information among the 10 individuals available in both adjacency matrix and edge list formats. Note that the network information is stored along with the attribute information in the same file. This type of file should still be opened/loaded using the attribute file icon.

*schlattr*.*rda* & *schlnet*.*rda*. A set of 100 respondents is randomly drawn from the Wave I public use data of the National Longitudinal Study of Adolescent to Adult Health (Add Health), available from the Interuniversity Consortium for Political and Social Research (ICPSR) web-site (http://www.icpsr.umich.edu/icpsrweb/DSDR/studies/21600). The *schlattr*.*rda* file includes sociodemographic information for 20 individual characteristics; and the group affiliation information for memberships in 5 kinds of organizations. The *schlnet*.*rda* file contains artificial network information that is simulated from an exponential family random graph (ERG) model [45, 46]. Note that unlike BSANet.rda this dataset stores attribute information in one file (schlattr.rda) and network information in another (schlnet.rda). Thus the former should be loaded/opened using the attribute file icon while the later using the network file icon.

*TwoCities*.*rda*. This dataset is excerpted, with permission, from the Social Capital Bench-mark Survey, which was collected by Professor Robert D. Putnam of the Saguaro Seminar: Civic Engagement in America, a project of the John F. Kennedy School of Government at Harvard University and numerous community foundations nationwide, and made available through the Roper Center for Public Opinion Research. The dataset contains 1,008 individuals from two cities in the United States: Bismarck, North Dakota, and Grand Rapids, Michigan. The sociodemographic information includes 4 individual characteristics–age, income, education level, and work time; and the group affiliation information includes membership in 18 voluntary organizations. There are also six other assorted variables.

*gss74_87*.*rda*. This dataset is excerpted from the General Social Survey, 1972–2012, available from both the Interuniversity Consortium for Political and Social Research (ICPSR) website (http://www.icpsr.umich.edu/icpsrweb/NACJD/studies/34802) and the NORC website (http://gss.norc.org/get-the-data/). The sociodemographic information includes the education level and occupational prestige scores of 13,865 respondents surveyed in 1974, 1975, 1977, 1978, 1980, 1983, 1984, 1986, and 1987; and the group affiliation information includes membership in 16 types of organizations.

## Results

In this section, we discuss the major capabilities of Blaunet for analyzing Blau space and use the example datasets to illustrate them. We begin by discussing a tool we developed for constructing Blau space. Researchers often have numerous sociodemographic variables at their disposal. However, not all of these are appropriate in constructing the Blau parameters. It is therefore desirable to have a principled method to identify those variables that can serve as salient dimensions for constructing a Blau space. This is the first capability of Blaunet and we discuss it in the “Salient dimension identification” subsection.

Before conducting the analysis, it is useful to visualize how the data “fits together”, how the individuals are distributed vis-à-vis the niches in Blau space, as well as how the network ties are distributed. Such visualization is also useful after the analysis since it sometimes clarifies certain patterns in the data. The challenge is in representing the three types of information. This is the second capability of Blaunet and we discuss it in the “Niche plot” subsection.

The main capability of Blaunet is the analysis of Blau space into Blau statuses. Based on the diverse types of information (i.e., sociodemographic, affiliation, and network) that can be processed, Blaunet can perform the analysis at three levels–nodal level, dyadic level, and meso level. As shown in Table 1, when a dataset contains only the sociodemographic information, Blaunet can only estimate statuses at the nodal level. At this level, Blaunet can generate a matrix indicating the list of individuals who are close to each focal individual in the Blau space using Blau bubble (proximity) analysis (see more details in the “Blau bubble (proximity) analysis” subsection).

**Table 1:** Blau statuses based on analytical levels and types of information.

		ANALYTICAL LEVELS		
		Nodal Level	Dyadic Level	Meso Level
**TYPES OF INFORMATION**	**Sociodemographic**	Blau bubble (proximity) analysis		
	**Sociodemographic, group Affiliation**	+Blau status for each individual	+Blau status for each pair of individuals	+Focal niche analysis, niche-by-niche analysis, bivariate correlation analysis
	**Sociodemographic, group Affiliation, network**	+Blau status for each individual through edges	+Blau status for each edge	+Network measures in bivariate correlation analysis

When sociodemographic information and group affiliation information are both available (e.g., similar to the example datasets *TwoCities*.*rda* and *gss74_87*.*rda*), Blau statuses at all three levels can be calculated. At the nodal level, Blaunet can indicate the Blau status of each individual in relation to the niches (e.g., outsider, insider, exclusive, manifolder, and member/nicher mixing status in each social entity). At the dyadic level, Blaunet can calculate the Blau status for each pair of individuals in relation to their niches (e.g., co-nicher, co-outsider, and Euclidean and Mahalanobis distance). And at the meso level, Blaunet can perform niche-by-niche analysis, focal niche analysis, and a bivariate correlation analysis for each social entity (see more details in the “Niche analysis” subsections).

When all three types of information are available (e.g., similar to the example datasets *BSANet*.*rda* and *schlattr*.*rda* & *schlnet*.*rda*), Blaunet can estimate some additional statuses for each level of analysis. At the nodal level, Blaunet can calculate the Blau status of each individual through existing edge(s) (e.g., spanner and number of niches spanned to). At the dyadic level, it can calculate Blau statuses of each edge (e.g., straddler and spanner). And at the meso level, Blaunet can add some network measures into the bivariate correlation analysis (see more details in the “Niche analysis” subsections).

One of the major goals of Blau space analysis has been to attempt to analyze niche movement over time [23]. The object of this analysis is to predict how each niche will move in a Blau space due to the competition among various social entities. Such niche dynamics analysis requires both sociodemographic and group affiliation information, but not necessarily network information. The question of niche dynamics is best addressed with longitudinal data but can also be approached with cross-sectional data. This is another capability of Blaunet and we discuss and give examples using both kinds of data in the “Niche dynamics analysis” subsection.

### Salient dimension identification

One of the limitations of Blau space analysis has been that there is no established way of identifying the dimensions that constitute it. All sociodemographic characteristics are potential Blau space dimensions, including both continuous characteristics such as age, years of education, income, and occupational prestige, as well as categorical measures such as gender, race, marital status, religion, and birthplace. However, which variables a researcher should choose as dimensions is currently determined purely by theoretical or substantive knowledge of the phenomena under study (e.g., theory of organizations, or substantive knowledge about a given area) with no principled method for quantitatively evaluating the choices. Researchers typically cite previous work or appeal to theoretically plausible variables that are known to influence association. This lack of a principled way of selecting dimensions can introduce bias into the analysis. It is possible to inadvertently omit an important dimension or to include a dimension that is not important in a given context. A way forward is to try to make the identification of salient dimensions more rigorous. This is one of the goals of Blaunet and is one of its most innovative features. From a list of theoretically plausible variables, Blaunet uses exponential random graph models [45] to identify variables that influence association probabilities and hence would be good candidates as dimensions to structure the Blau space. However, the analyst is advised to bear in mind that these tools are a supplement to researcher judgment rather than a replacement of it. Some theoretical guidance should still be used in selecting the appropriate dimensions.

**Fig 3:**
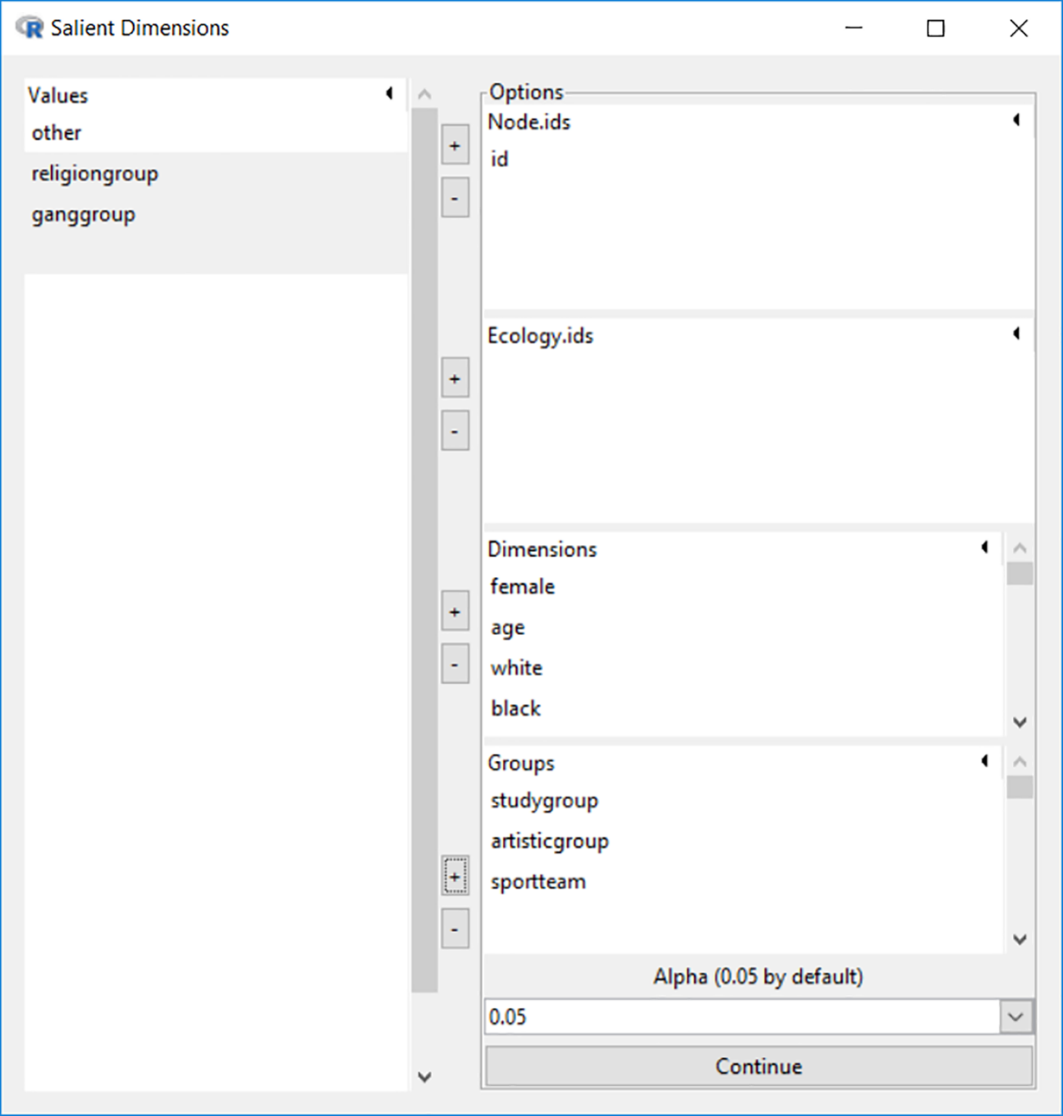
Graphic interface for salient dimension suggestion.

The salient dimension identification function is the first item under the “Analysis” menu. As shown in Fig 3, the example dataset *schlattr*.*rda* & *schlnet*.*rda* is loaded and all 20 sociodemographic variables are screened to identify the salient dimensions for 3 groups–study group, artistic group, and sports team. An automated iterative procedure is implemented in Blaunet for selecting a set of dimensions that is appropriate for constructing a Blau space for the population under study.

Theoretically, salient dimension identification is based on two standards. First, at the individual level, the selected set of salient dimensions should help explain the membership of an individual in the *m* social entities. This function is accomplished by running a series of *m* logistic regression models:

$$P(Y_{o\in (1,m)}=1)=\frac{1}{1+e^{-(\alpha +\sum \beta x_{ik})}}.$$ (1)

In Eq (1), *P*(*Y*_*o*∈(1,*m*)_ = 1 is the probability that an individual is a member of the *o*th social entity, given a linear combination *x* of *k* sociodemographic traits. It is not surprising that a few dimensions might be salient for some entities but not for the others. We combine the sociodemographic traits that predict (e.g., at the significance level of 0.05 by default) the membership of at least one social entity into a preliminary list.

Second, at the dyadic level, the homophily on salient dimensions is expected to explain the co-membership of a pair of individuals in *m* social entities. This function is accomplished by estimating a series of *m* exponential family random graph (ERG) models [47]:

$$P(Y_{o\in (1,m)}=y_{o\in (1,m)})=\frac{1}{\kappa }e^{\theta_{hcav}y_{ij}I(x_{i}=x_{j})+\theta_{hcov}y_{ij}{|x}_{i}-x_{j}|+\theta_{net}{{net}_{ij}y}_{ij}}.$$ (2)

**Fig 4:**
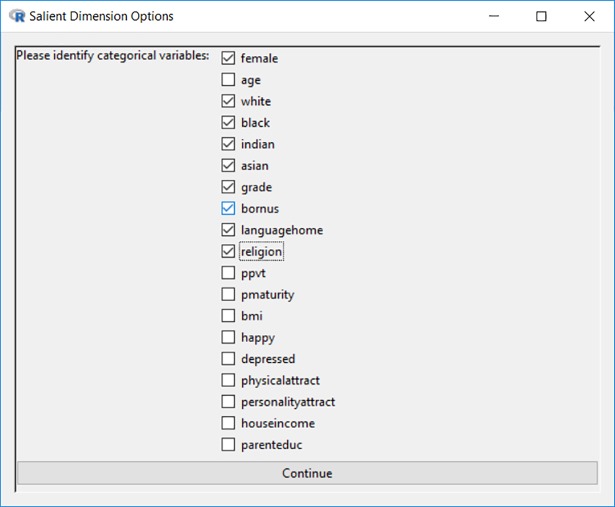
Identifying categorical variables among the set of dimensions.

In Eq (2), *Y*_*o*∈(1,*m*)_ is the symmetric co-membership matrix for the *o*th social entity with the state *y*_*o*∈(1,*m*)_. The *x*_*i*_ and *x*_*j*_ are the vectors of statistics on each sociodemographic trait for a pair of individuals *i* and *j*. As shown in Fig 4, categorical variables are differentiated from continuous variables. For categorical variables, this homophily effect is estimated as *θ*_*hcav*_. This is implemented by using the nodematch term in the ergm package [46] of statnet [48, 49]. For continuous variables, this homophily effect is estimated as *θ*_*hcov*_. This is implemented by using the absdiff term in the ergm package [46] of statnet [48, 49].

**Fig 5:**
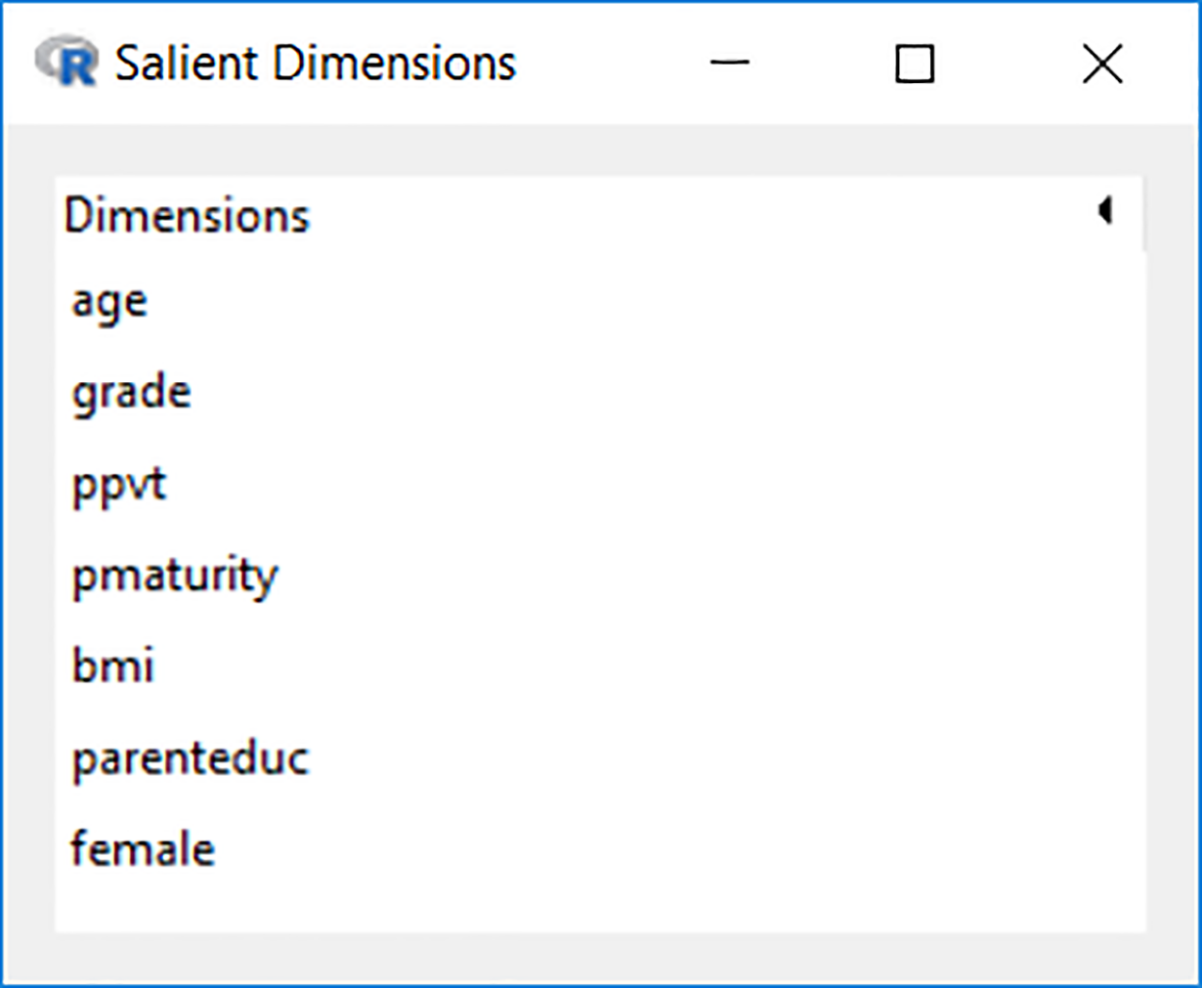
Results for identified salient dimensions.

When a network dataset is available and loaded into memory, the homophily effect of social ties on the co-membership state will be estimated as *θ*_*net*_. The denominator *κ* is the normalizing factor that ensures that Eq (2) is a legitimate probability distribution. All the sociodemographic characteristics in the primary list through *m* logistic regression models based on Eq (1) enter the *m* ERG models based on Eq (2), and those found to be insignificant (e.g., *p* > = 0.05 by default) in all *m* ERG models are cut. As shown in Fig 5, there are 7 remaining sociodemographic characteristics that make up the finalized list of salient dimensions identified by Blaunet. Researcher-selected dimensions that also appear on this list can be viewed as being independently supported, though this Blaunet routine is a heuristic and should be supplemented by sociological theory and content-level expertise.

Note that, as of now, only continuous sociodemographic variables can be used as dimensions for constructing Blau space. While prior Blau space analysis has used categorical variables as dimensions [1], this required including them as proportions of an organization that falls into a particular category (e.g., proportion female). While this method works for organizations to a degree, it is ineffective for individuals, who occupy discrete values. The standard practice in Blaunet is to input categorical variables during the modelling stage of the analysis.

### Niche plot

Blaunet provides the niche plotting feature introduced by McPherson [1]. Niches of social entities can be represented in the Blau space as boxes (in 2-dimensions) or cuboids (in 3-dimensions) overlaying the points that indicate the individuals who belong to them.

**Fig 6:**
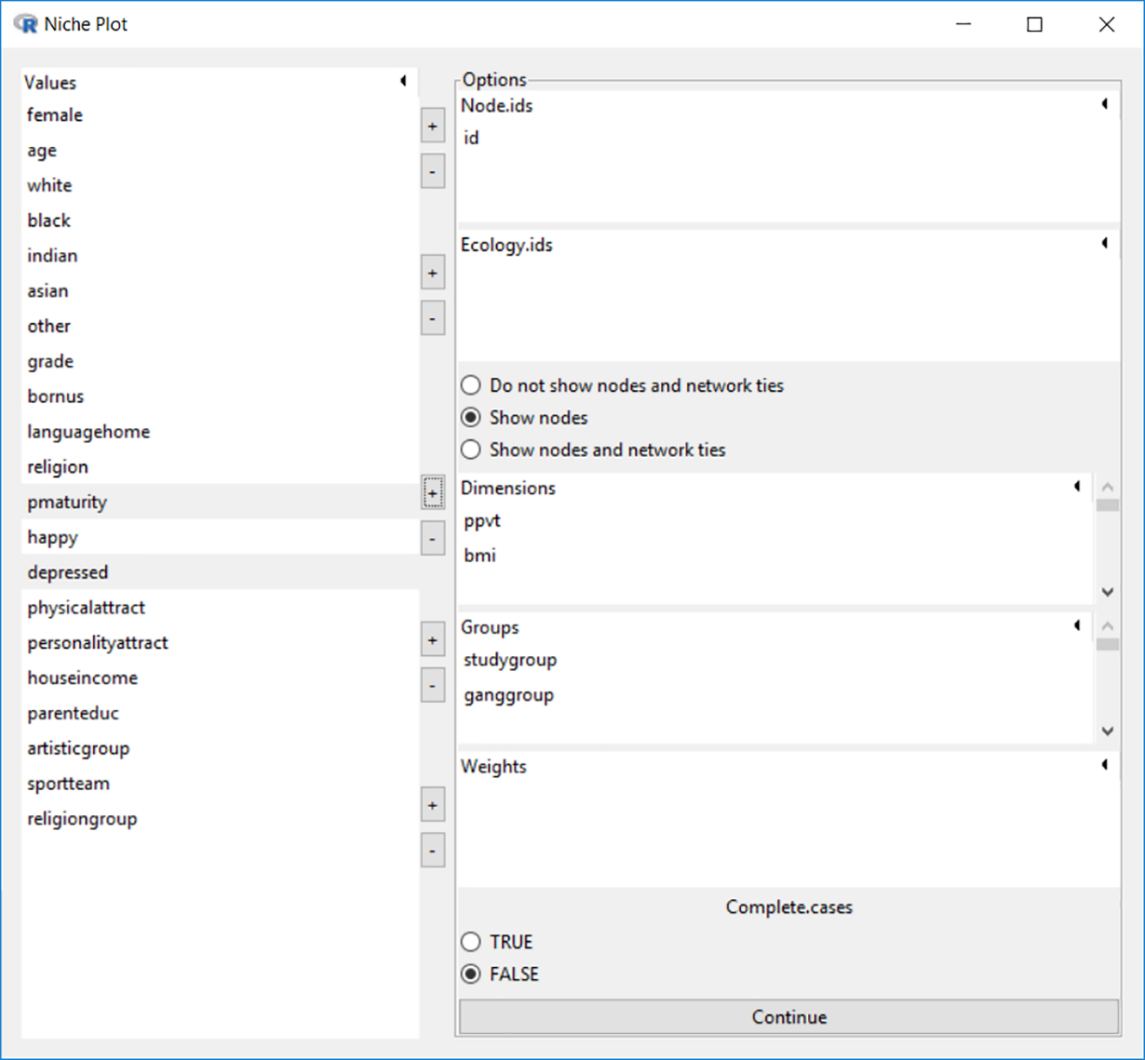
Graphic interface for niche plot.

The niche plot function is the second item under the “Analysis” menu. As shown in Fig 6, the example dataset *schlattr*.*csv* & *schlnet*.*csv* is loaded and the Peabody Picture Vocabulary Test score (PPVT, the horizontal axis) and Body Mass Index (BMI, the vertical axis) are used as the two salient dimensions for the study group and gang group.

**Fig 7:**
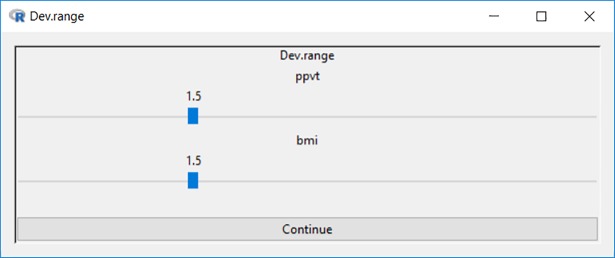
Setting deviation range for niche plot.

The niche for each group is centered on the mean value of each dimension and extends 1.5 standard deviations (following the default breadth set by McPherson [1], but configurable in Blaunet) above and below the mean, as shown in Fig 7.

**Fig 8:**
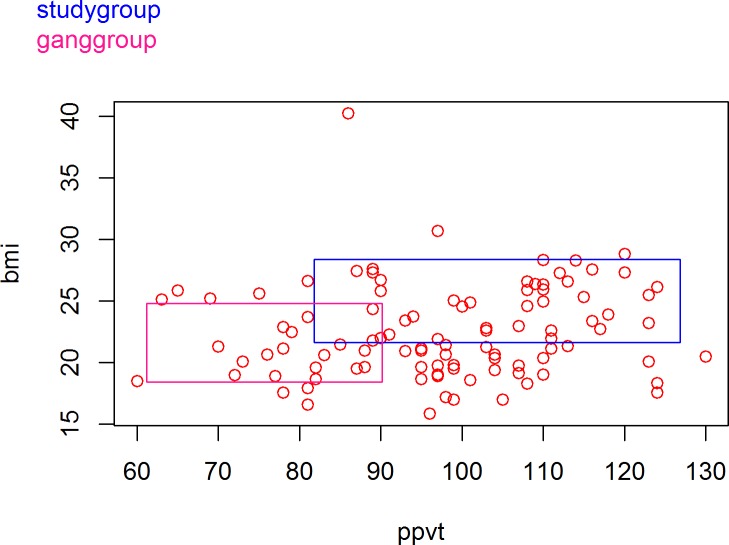
Niches of study group and gang group along two dimensions.

The area inside each group’s box in Fig 8 is its niche in the two-dimensional space, and the intersection of the boxes is the region in which the two groups are competing for the same type of members. The members of the study group (blue box) tend to have higher PPVT scores (82 to 127) than those of the gang group (61 to 90). On the other hand, the gang members (pink box) tend to have lower BMI (18 to 25) than the study group members (22 to 28). Individuals are colored as red circles based on their values on the two dimensions. Individuals located in one or more niches are defined as insiders, compared to outsiders who fall outside of any niche. Insiders can be further categorized into two groups based on the number of niche(s) they are in–exclusives, who are located only in one niche and manifolders, who are located in more than one niche (inside the intersecting region(s) of Blau space). When network data is available and loaded into memory, Blaunet will automatically detect it and provide an option for users to display the network edges in the niche plot.

**Fig 9:**
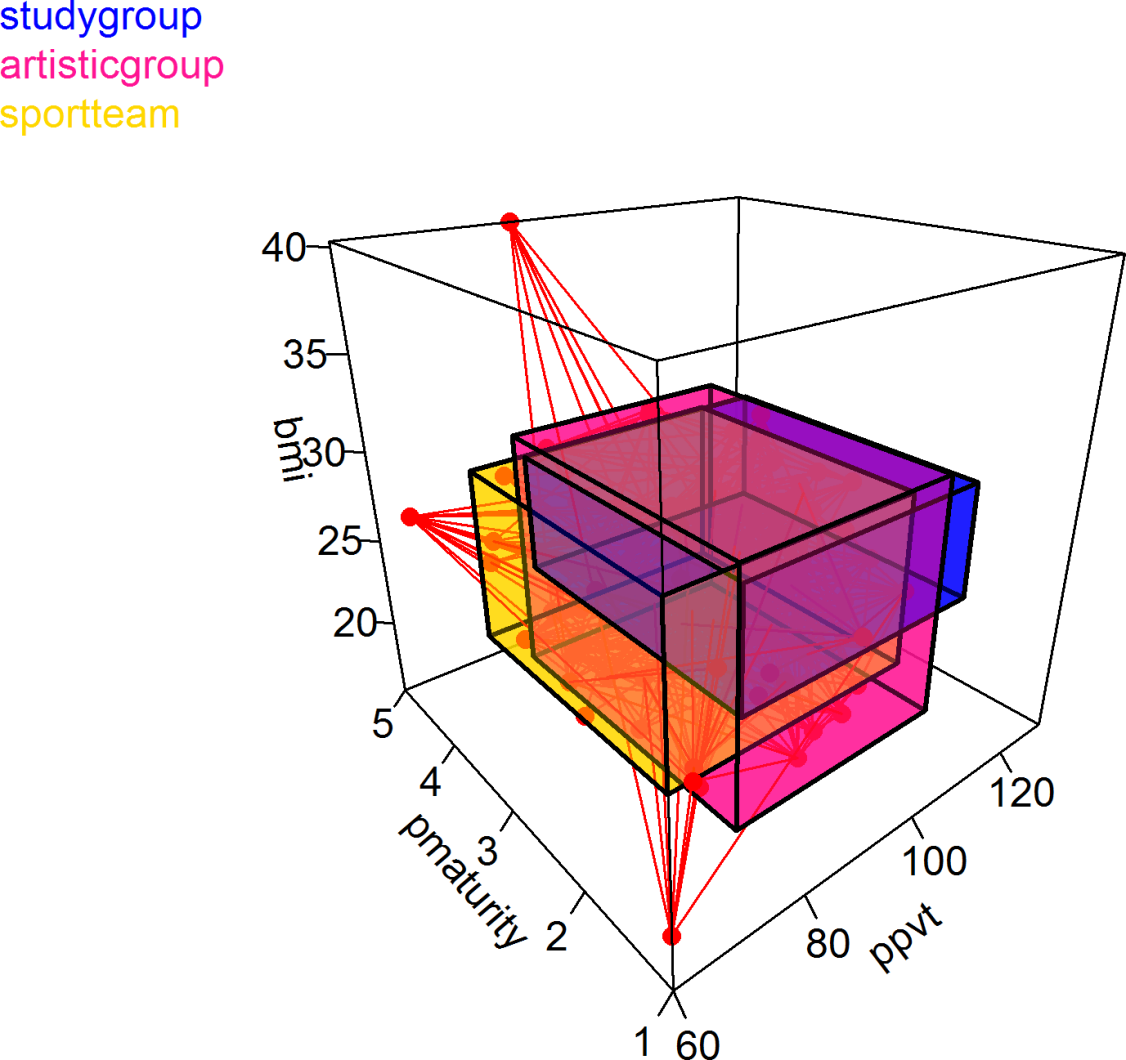
Niches of study group, artistic group, and sport team along three dimensions.

Blaunet allows for a maximum of three salient dimensions to be displayed in the Blau space. As shown in Fig 9, the example dataset *schlattr*.*csv* & *schlnet*.*csv* is loaded and Physical Maturity (the left axis), PPVT (the right axis), and BMI (the vertical axis) are used as the three salient dimensions for the study group, artistic group, and sport team. The cuboids colored in blue, pink, and yellow are the niches of study group, artistic group, and sport team in the three-dimensional space, respectively. The individuals are represented as red spheres and the option to display the network edges is selected. The three-dimensional plot is rotatable to detect details about how social entities are distributed along the three salient dimensions in the Blau space.

### Blau bubble (proximity) analysis

Thus far, we considered the use of Blaunet to calculate metrics that involve organizational membership. By contrast, Blau bubbles are not based on organizational competition and their niches, nor do they require the analyst to take organizational membership information into account. Instead they are based on one’s proximity to other actors in Blau space.

Proximity in terms of network ties has been long recognized as a conduit for social influence [50]. A person (ego) is likely to influence and be influenced by their friends, co-workers, and lovers on a variety of attitudes and behaviors; and there is now a large literature that attempts to disentangle influence effects from selection effects in social networks [51–54]. Recent work has attempted to look for social influence beyond traditional network contacts and to incorporate other actors who are part of the ego’s social context [55–57]. One way this is done is to include sociodemographically similar individuals with whom the ego shares a physical environment (e.g. school, workplace, neighborhood) but who would not necessarily be considered “friends” by the ego. Such individuals have been termed *familiar others* [41] and are captured using Blau bubble analysis. Familiar others have been shown to exert influence on a range of ego’s behaviors [41, 58]. Though the influence occurs through different mechanisms than in network ties, such as social comparison, behavioral modeling, and observational learning [58]. Suh, Shi, and Brashears [41] applied Blau bubble analysis to the Add Health data to identify possible familiar others, and found that they constitute a salient source of social influence for an individual's smoking behavior, which is quite distinct from the influence through network relations. Behler et al. [58] found that the unhealthy weight-related behaviors of familiar others strongly predicted the ego’s own weight behavior and that this relationship is stronger than that between the ego and their direct network ties. Blau bubble analysis thus provides a probabilistic approach to network analysis and social influence when conventional network information is unavailable. It can also be used to capture weaker forms of contact, such as casual contacts, that are typically missed on more conventional network inventories.

Blau bubble (proximity) analysis relies only on sociodemographic information [41]. Each individual is positioned with unique coordinates along the salient sociodemographic dimensions in the Blau space (e.g., the red circles in a two-dimensional space as shown in Fig 8 or the red spheres in a three-dimensional space as shown in Fig 9). The more similar two individuals are on the set of salient sociodemographic parameters that define the Blau space, the more proximate they are in the hyperspace coordinate system, and the more likely they are to associate with and be influenced by one another. Just like the niche of a social entity marks its boundary for recruiting potential members, we identify a Blau bubble for each individual as being comprised of his or her similar others situated within a specific distance from the location of focal individual in the Blau space.

Blau bubbles are calculated in the following way. In Blaunet, the Euclidean distance of each focal individual *i* from all other individuals along *k* sociodemographic dimension within the Blau space is defined as

$${Dis}_{ij}=\sqrt{\frac{\sum_{p=1}^{k}{\left(x_{pi}^{\prime }-x_{pj}^{\prime }\right)}^{2}}{k}},$$ (3)

where *x*′ indicates the value on a given dimension and *k* is the number of dimensions in Blau space. For categorical dimensions, a pair of individuals that belong to the same category (e.g., both are African-Americans or married) are assigned a value of zero (i.e., $x_{pi}^{\prime }-x_{pj}^{\prime }=0$), and otherwise are assigned a value of one (i.e., $x_{pi}^{\prime }-x_{pj}^{\prime }=1$). Continuous variables are standardized to the unit interval as

$$x_{pi}^{\prime }=\frac{x_{pi}-min(x_{pi})}{\max\left(x_{pi}\right)-min(x_{pi})},$$ (4)

where *x* is the observed value on a given dimension. The conversion enables the score *x*′ in Eq (3) to take any value between zero and one. Consequently, all dimensions range from zero to one. Thus, for *n* individuals in the Blau space, the distance from the focal individual to any other individual along the *k* dimensions is calculated *n*-1 times and a distance matrix of dimensions *n* by *n* is generated.

Let us begin with the example dataset *BSANet*.*rda*, which includes attribute, affiliation, and network data. The attribute data includes individual names as node ids, three salient dimensions (i.e., age, income, education), and two group affiliation indicators (i.e., conservative and liberal) for 10 persons in two ecologies (i.e., New York and San Francisco). The attribute data in Table 2 can be displayed by clicking the first item under the “Browse” menu.

**Table 2:** Attribute data in the *BSANet*.*rda*.

	person	city	age	income	educ	grp_conservative	grp_liberal
1	John	New York	24	5	7	1	1
2	Emma	New York	38	1	3	0	1
3	Mary	New York	58	2	7	1	0
4	Amir	New York	47	4	2	0	0
5	Mark	New York	33	4	4	0	0
6	Bryan	San Francisco	23	0	7	1	0
7	Wendy	San Francisco	28	6	0	1	1
8	Aude	San Francisco	22	1	4	1	1
9	Mona	San Francisco	29	7	5	1	1
10	Bruce	San Francisco	41	5	7	1	1

The network part of *BSANet*.*rda* gives the network relational information among the 10 individuals. In the adjacency matrix shown in Table 3, a value of 1 indicates that there is a directed edge from the individual in the row (ego) to another individual in the column (alter) while 0 indicates otherwise. In this case there is no edge between any individual in New York City and that in San Francisco. The adjacency matrix in Table 3 can be displayed by clicking the second item under the “Browse” menu.

**Table 3:** Network data in the *BSANet*.*rda*.

Ego	John	Emma	Mary	Amir	Mark	Bryan	Wendy	Aude	Mona	Bruce
John	0	1	1	0	0	0	0	0	0	0
Emma	1	0	1	0	0	0	0	0	0	0
Mary	1	1	0	0	0	0	0	0	0	0
Amir	0	0	0	0	1	0	0	0	0	0
Mark	0	0	0	1	0	0	0	0	0	0
Bryan	0	0	0	0	0	0	1	1	0	0
Wendy	0	0	0	0	0	1	0	1	0	0
Aude	0	0	0	0	0	1	1	0	0	0
Mona	0	0	0	0	0	0	0	0	0	1
Bruce	0	0	0	0	0	0	0	0	1	0

**Fig 10:**
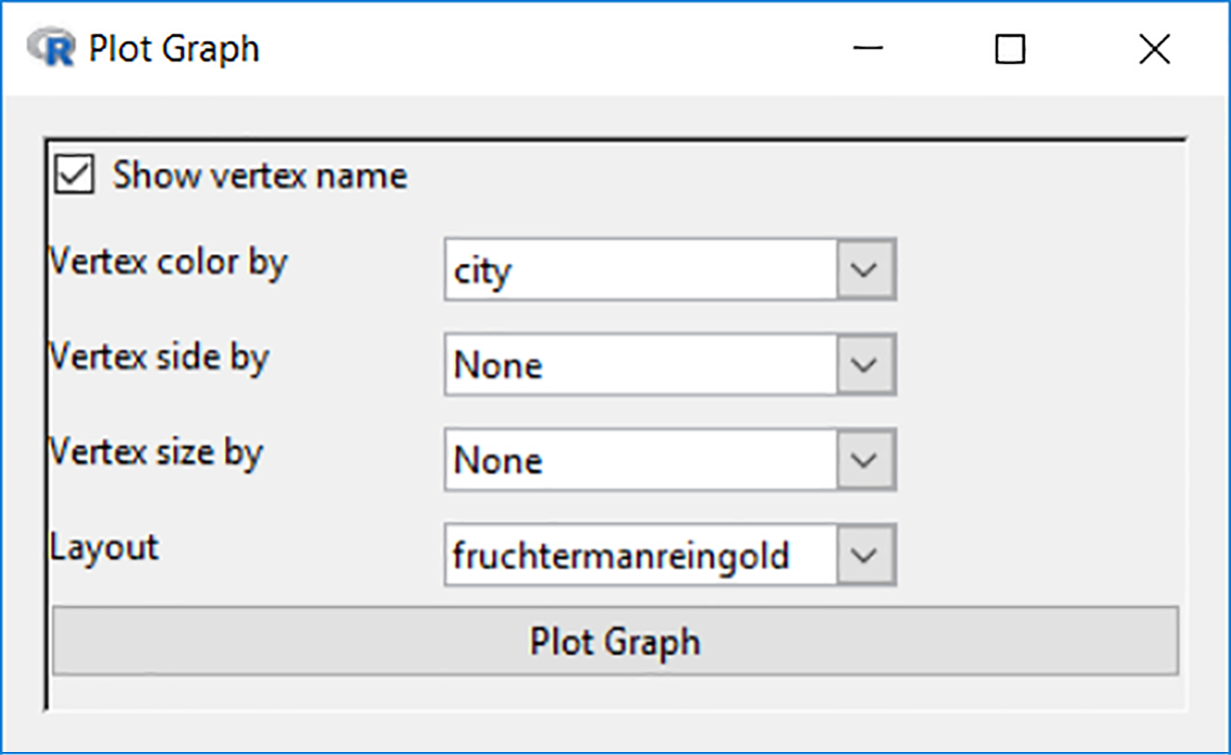
Options for plotting network graph.

The adjacency matrix can be plotted as a network graph by clicking the first item under the “Graph” menu. As shown in Fig 10, Blaunet users can choose whether vertex names/labels will appear in the graph, how to define the vertex color, sides (e.g., starts from a minimum of 3-sided or a triangle), and size, as well as what type of layout will be applied.

**Fig 11:**
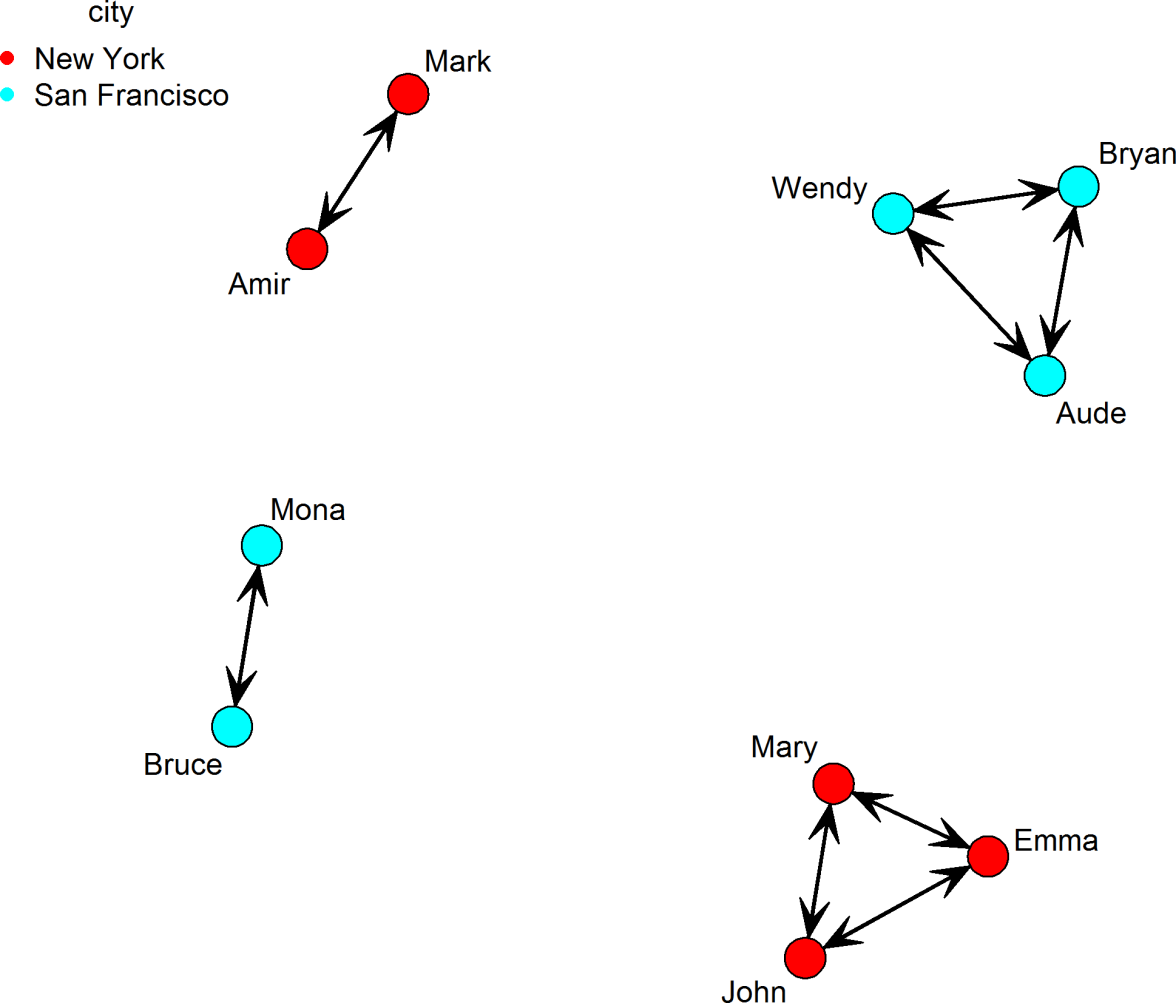
The network graph of *BSANet*.*rda*.

Red circles in Fig 11 are used to indicate individuals in New York City (the first five egos listed in the adjacency matrix) while cyan circles are used to indicate those in San Francisco (the last five).

**Fig 12:**
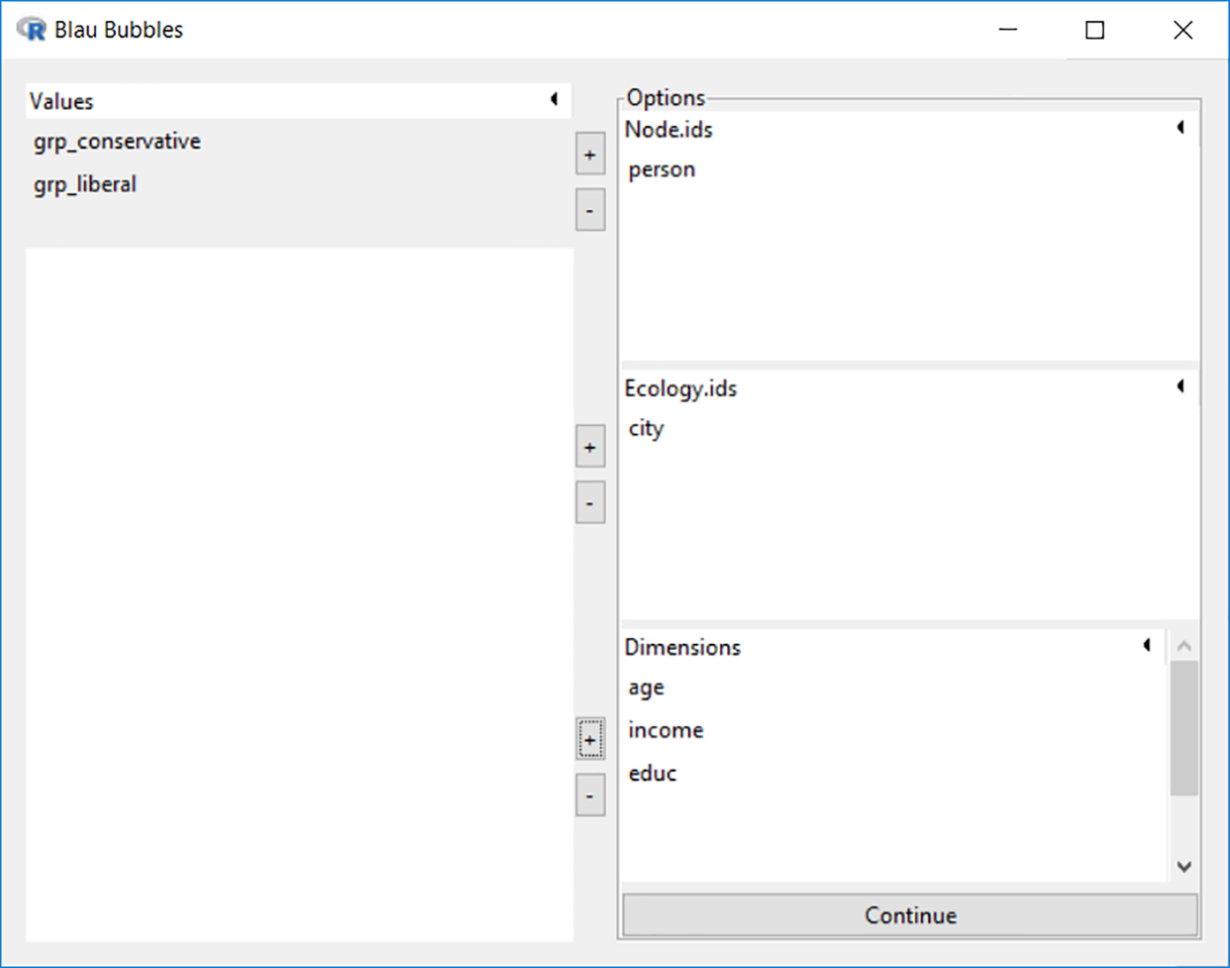
Interface for Blau bubble analysis.

The Blau bubble analysis menu is accessed by clicking the last item under the “Analysis” menu. As shown in Fig 12, all 3 dimensions (*age*, *income*, *educ*) in *BSANet*.*rda* are utilized to perform the Blau bubble (proximity) analysis, while the *city* variable is selected to identify the ecology.

**Fig 13:**
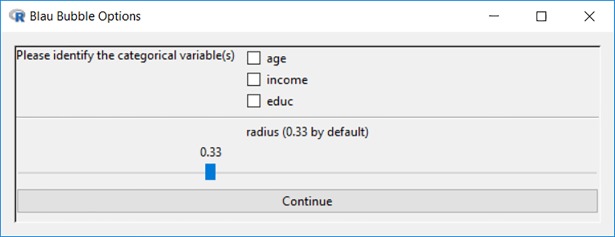
Options for Blau bubble analysis.

Blaunet users can select a cutoff between 0 and 1 as the radius to set the boundary for one’s possible similar others. More specifically, those falling within the radius of the focal individual will be included within his or her Blau bubble. When the radius is set to 0, only those whose sociodemographic characteristics completely match with the focal individual will be classified as possible similar others. At the other extreme, when the radius is set as 1, all individuals in the Blau space will be captured. By default, Blaunet assigns a radius of 0.333 for building the Blau bubbles, as shown in Fig 13.

**Fig 14:**
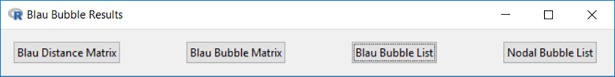
Results from Blau bubble analysis.

Blaunet presents at least three types of results for Blau bubble (proximity) analysis. When network information is loaded in Blaunet, an additional “Blau Bubble list” becomes available, as shown in Fig 14.

The first result is the “Blau Distance Matrix” as shown in Table 4, with a score of 0 on *Dis*_*ij*_ indicates perfect similarity between individuals *i* and *j*, and a score of 1 indicates perfect dissimilarity.

**Table 4:** Distance matrix among individuals from *BSANet*.*rda*.

ego	John	Emma	Mary	Amir	Mark	Bryan	Wendy	Aude	Mona	Bruce
John	0.0000	0.5178	0.5988	0.5594	0.2981	0.4127	0.5867	0.4136	0.2467	0.2726
Emma	0.5178	0.0000	0.4675	0.2981	0.2729	0.4166	0.5070	0.2695	0.5412	0.4690
Mary	0.5988	0.4675	0.0000	0.4779	0.4992	0.5850	0.8208	0.6335	0.6431	0.3682
Amir	0.5594	0.2981	0.4779	0.0000	0.2786	0.6535	0.3838	0.4992	0.4536	0.4314
Mark	0.2981	0.2729	0.4992	0.2786	0.0000	0.4425	0.3775	0.3039	0.2686	0.2907
Bryan	0.4127	0.4166	0.5850	0.6535	0.4425	0.0000	0.7646	0.2613	0.6081	0.5034
Wendy	0.5867	0.5070	0.8208	0.3838	0.3775	0.7646	0.0000	0.5368	0.4209	0.6194
Aude	0.4136	0.2695	0.6335	0.4992	0.3039	0.2613	0.5368	0.0000	0.5141	0.5128
Mona	0.2467	0.5412	0.6431	0.4536	0.2686	0.6081	0.4209	0.5141	0.0000	0.3024
Bruce	0.2726	0.4690	0.3682	0.4314	0.2907	0.5034	0.6194	0.5128	0.3024	0.0000

When the default radius of 0.333 is applied, as shown in Table 4, the distance between John and Emma is 0.52, and thus Emma is outside of John’s Blau bubble; the distance between John and Bruce is 0.27, and thus Bruce is inside of John’s Blau bubble. The “Blau Bubble Matrix” is shown in Table 5.

**Table 5:** Blau bubble matrix among individuals from *BSANet*.*rda*.

ego	John	Emma	Mary	Amir	Mark	Bryan	Wendy	Aude	Mona	Bruce
John	0	0	0	0	1	0	0	0	1	1
Emma	0	0	0	1	1	0	0	1	0	0
Mary	0	0	0	0	0	0	0	0	0	0
Amir	0	1	0	0	1	0	0	0	0	0
Mark	1	1	0	1	0	0	0	1	1	1
Bryan	0	0	0	0	0	0	0	1	0	0
Wendy	0	0	0	0	0	0	0	0	0	0
Aude	0	1	0	0	1	1	0	0	0	0
Mona	1	0	0	0	1	0	0	0	0	1
Bruce	1	0	0	0	1	0	0	0	1	0

Blaunet also provides a “Nodal Bubble list”, which gives the summary of the individuals in one’s Blau bubble (“co_bubble”) as well as who they are (“co_bubble_list”). When network information is available and loaded in memory as in this case, the summary table also includes four additional columns. The first two additional columns (“degree” and “alter_list”) indicate how many alters each individual connects to as well as who they are. The second two additional columns (“coincidence” and “coincidence_list”) provide the number of overlaps between the Blau bubble list and the alter list as well as who they are. As shown in Table 6, Amir has Emma and Mark in his Blau bubble and Mark in his alter list, and thus Mark is the only one appearing in both lists.

**Table 6:** Blau bubble summary for individuals from *BSANet*.*rda*.

person	co_bubble	co_bubble_list	degree	alter_list	coincidence	coincidence_list
John	3	Mark Mona Bruce	2	Emma Mary	0	
Emma	3	Amir Mark Aude	2	John Mary	0	
Mary	0		2	John Emma	0	
Amir	2	Emma Mark	1	Mark	1	Mark
Mark	6	John Emma Amir Aude Mona Bruce	1	Amir	1	Amir
Bryan	1	Aude	2	Wendy Aude	1	Aude
Wendy	0		2	Bryan Aude	0	
Aude	3	Emma Mark Bryan	2	Bryan Wendy	1	Bryan
Mona	3	John Mark Bruce	1	Bruce	1	Bruce
Bruce	3	John Mark Mona	1	Mona	1	Mona

### Niche analysis

In Blaunet, five elements are required to generate an object of the "Blau" class for niche analysis, including:

1. node id–a unique identifier for each individual. When sociodemographic and network information are both available, this is the key that links these two types of information;
2. salient dimensions–selected sociodemographic attributes which comprise the Blau parameters for the model;
3. group affiliation indicators–the membership of individuals in social entities such as groups or organizations;
4. deviation range–indicators for standard deviation around the mean in each salient dimension to include in the niche (default is 1.5);
5. complete case option–a Boolean setting indicating whether individuals with at least one missing value should be dropped before proceeding with niche analysis (default is “false”).

There are also three optional elements, including:

6 ecology–identifies when individuals are partitioned into sub-samples (e.g., geospatial locations as in the example datasets *BSANet*.*rda* and *TwoCities*.*rda* or time points as in the example dataset *gss74_87*.*rda*). This is important because social entities such as organizations compete for members in the same ecology. For example, a club in one city cannot compete for members who live in a different city.
7 weights–uses a weight variable for each individual when individuals are sampled from a population and the standard deviation of the Blau parameters needs to be adjusted (default is 1 –everyone has the same weight);
8 network–when interpersonal network relational information is available and loaded in memory.

**Fig 15:**
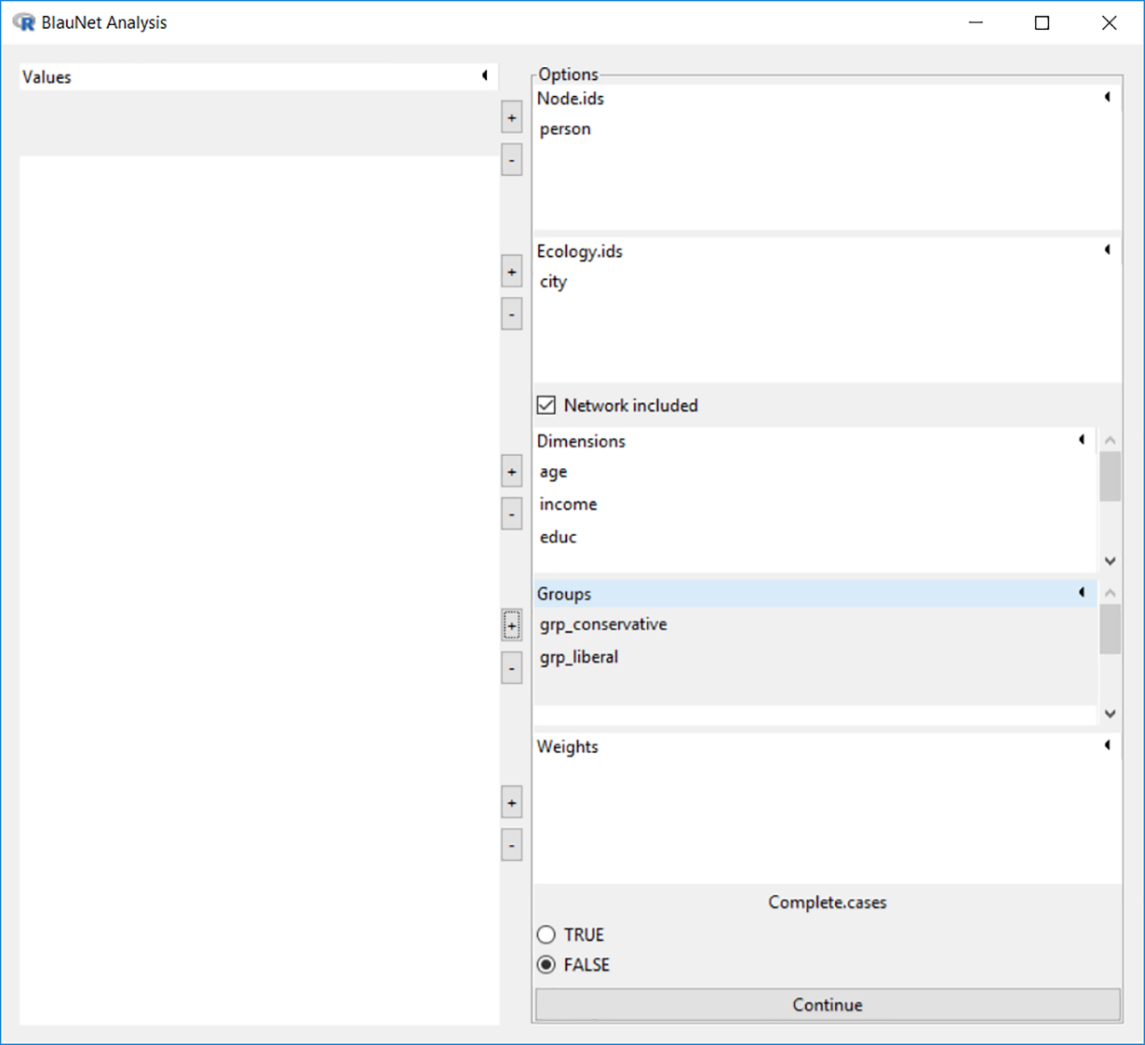
Interface for niche analysis.

The niche analysis function is the third item under the “Analysis” menu. Let us consider an example using the dataset *BSANet*.*rda*, illustrated in Fig 15. The variable *person* is selected as the node id, the variable *city* as the ecology id, all 3 sociodemographic measures (*age*, *income*, *educ*) are selected as the salient dimensions, memberships in a liberal or conservative organization (*grp_conservative*, *grp_liberal*) are selected as group affiliation indicators, and since network relational information is available, the “Network included” box is checked.

**Fig 16:**
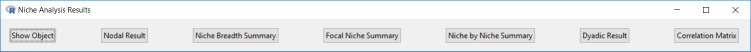
Niche analysis results.

After setting the deviation range for each salient dimension, niche analysis provides at least 6 types of results, as shown in Fig 16. When network information is loaded in Blaunet, an additional “Dyadic Result” is also available.

In “Niche Breadth Summary”, following McPherson [1], Blaunet calculates niche breadth as the mean ± 1.5 standard deviation (by default) for each specific sociodemographic dimension as shown in Fig 8 and Fig 9. As shown in Table 7, members of the New York conservative group (John and Mary) have a mean income of 3.50 and a standard deviation of 2.12. Thus the niche breadth for the conservative group in New York City ranges from 0.32 to 6.68 on the dimension “income”. Note that when a salient dimension can only take positive values (e.g., all three dimensions in this case), if the result of the “mean minus 1.5 times the standard deviation” calculation is negative then we force the low bound to be 0 (e.g., this is the case with the low bounds of income for the niche of the New York liberal group as well as the niche of the San Francisco conservative group. It is also the case for the parameter education level for the niche of the San Francisco conservative group).

**Table 7:** Niche breadth summary for the “Blau” object generated from *BSANet*.*rda*.

GROUPS	age	income	educ	ecologyNames
grp_conservative	4.94–77.06	0.32–6.68	7.00–7.00	New York
grp_liberal	16.15–45.85	0.00–7.24	0.76–9.24	New York
grp_conservative1	17.25–39.95	0.00–8.47	0.28–8.92	San Francisco
grp_liberal1	18.06–41.94	0.81–8.69	0.00–8.42	San Francisco

At the nodal level, Blaunet users can browse the “Nodal Result” for each individual across all niches in the Blau space. In Table 8, the 3rd column “TotalOrgs” shows the total number of social entities that each individual is in, with the lower bound equaling to 0 and the upper bound equaling the maximum number of social entities populating the ecology. The 4th column “Niches” provides details about how many niches each individual is in, or how many social entities are competing for that individual (irrespective of actual membership). A value of 0 indicates that an individual is an outsider, that of 1 indicates that the individual is an exclusive (suggesting an organizational monopoly), and a value of 2 or more indicates that the individual is a manifolder (i.e., belongs to multiple niches and is a potential recruit for several social entities). The 5th column “NicheList” provides a list indicating which niche(s) each individual belongs to. The 6th to 8th columns indicate whether each individual is an outsider, insider & exclusive, and insider & manifolder (0 = no and 1 = yes).

**Table 8:** The first part of nodal output for the “Blau” object generated from *BSANet*.*rda*–New York city.

1	2	3	4	5	6	7	8	9	10
nodeId	ecologyId	TotalOrgs	Niches	NicheList	Outsider	Insider_Exclusive	Insider_Manifolder	grp_conservative	grp_liberal
John	New York	2	2	1 2	0	0	1	1	1
Emma	New York	1	1	2	0	1	0	0	1
Mary	New York	1	1	1	0	1	0	1	0
Amir	New York	0	0		1	0	0	0	0
Mark	New York	0	1	2	0	1	0	0	0

In Table 9, the 13th and 14th columns show a mixing member/nicher status for each social entity (i.e., neither member nor nicher, non-member but in niche, member not in niche, or member & nicher). The last two columns “Spanner” and “NumSpannedTo” only show up when the network information is available and loaded in memory. An individual is a spanner if he or she is outside the niche of a particular social entity but another individual he or she has tied to is an insider, and thus he or she can span to the niche of that social entity through this tie. Therefore, the column “Spanner” indicates whether each individual is a spanner to other social entities through his or her existing ties (0 = no and 1 = yes) and the column “NumSpannedTo” indicates how many social entities the individual has spanned to through ties to other individuals. For example, as shown in the 11th and 12th columns, Emma is an outsider of the conservative group niche but an insider of the liberal group niche, and Mary is an insider of the conservative group niche but an outsider of the liberal group niche. Note that there is a mutual tie between Emma and Mary. Thus, Emma has spanned to the niche of the conservative group through her tie to Mary, and Mary has spanned to the niche of the liberal group through her tie to Emma.

**Table 9:** The second part of nodal output for the “Blau” object generated from *BSANet*.*rda*–New York city.

11	12	13	14	15	16
grp_conservative_niche	grp_liberal_niche	grp_conservative_mem.niche	grp_liberal_mem.niche	Spanner	NumSpannedTo
1	1	Member & nicher	Member & nicher	0	0
0	1	Neither member nor nicher	Member & nicher	1	1
1	0	Member & nicher	Neither member nor nicher	1	1
0	0	Neither member nor nicher	Neither member nor nicher	1	1
0	1	Neither member nor nicher	Non-member but in niche	0	0

The analytical outputs at the dyadic level, i.e. “Dyadic Result”, are shown in Table 10. The column “CoNicher” indicates whether a pair of individuals are insiders of the same niche (e.g., Emma and John are both insiders of the liberal group niche; 0 = no and 1 = yes). The column “CoOutsider” indicates that a pair of individuals are outsiders of any niche (and none is found in this case; 0 = no and 1 = yes). The column “Straddler” indicates that one node is an insider of at least one niche while the other is an outsider of any niche (e.g., Mark is an insider of the liberal group niche and Amir is an outsider of any niche; 0 = no and 1 = yes). The last two columns give the Euclidean distance and the Mahalanobis distance (i.e., Euclidean distance standardized by the unit of measurement) between a pair of individuals. When the network information is available and is loaded in memory, the column “Spanner” will show up and indicate how many different niche(s) an edge enables the two enclosed nodes to span (0 = no and 1 = yes). For example, for the mutual edges between Emma and Mary, Emma is an exclusive of the liberal group niche and Mary is an exclusive of the conservative group niche, and thus both of them have spanned to each other’s niche.

**Table 10:** Dyadic output for the “Blau” object generated from *BSANet*.*rda*.

Ego	Alter	CoNicher	CoOutsider	Straddler	Spanner	EucDist	MahalanobisDist
Amir	Mark	0	0	1	0	14.14214	1.576603
Emma	John	1	0	0	1	15.09967	2.801218
Emma	Mary	0	0	0	2	20.42058	2.771355
John	Emma	1	0	0	1	15.09967	2.801218
John	Mary	1	0	0	1	34.1321	2.661715
Mark	Amir	0	0	1	0	14.14214	1.576603
Mary	Emma	0	0	0	2	20.42058	2.771355
Mary	John	1	0	0	1	34.1321	2.661715
Aude	Wendy	1	0	0	1	8.774964	1.880606
Aude	Bryan	1	0	0	1	3.316625	1.0905
Bruce	Mona	1	0	0	1	12.32883	2.827745
Bryan	Wendy	0	0	0	2	10.48809	2.784402
Bryan	Aude	1	0	0	1	3.316625	1.0905
Mona	Bruce	1	0	0	1	12.32883	2.827745
Wendy	Bryan	0	0	0	2	10.48809	2.784402
Wendy	Aude	1	0	0	1	8.774964	1.880606

At the meso level, Blaunet outputs the “Niche by Niche Summary” for each ecology as shown in Table 11. For the first matrix in columns 3 and 4, the diagonal elements contain the number of individuals exclusive to the niche of each social entity and the off-diagonal elements correspond to the number of individuals overlapped by the other niches for each ecology. For example, for the New York City ecology, the conservative group has one exclusive in its niche (i.e., Mary), and the liberal group has two exclusives in its niche (i.e., Emma and Mark), and these two groups have one individual overlapped by both niches (i.e., John). The second matrix in columns 5 and 6, which is referred to as the matrix of competition coefficients in McPherson [1], is generated from the first niche-by-niche matrix by dividing its elements in each (row) social entity by the number of nichers in the corresponding (row) social entity (which will be shown as the column “NicheMem” in Table 12). Note that the matrix of competition coefficients might not be symmetric. The last two columns give the mean and standard deviation of the competition coefficients, calculated from the second matrix, for each ecology.

**Table 11:** Niche by niche summary for the “Blau” object generated from *BSANet*.*rda*.

Ecology	Org.Niche	grp_conservative	grp_liberal	grp_conservative_CC	grp_liberal_CC	AVG_CC	STD_CC
New York	grp_conservative	1	1	0.5	0.5	0.5	0.14
New York	grp_liberal	1	2	0.33	0.67	0.5	0.14
San Francisco	grp_conservative	1	2	0.33	0.67	0.5	0.14
San Francisco	grp_liberal	2	2	0.5	0.5	0.5	0.14

**Table 12:** Focal niche summary for the “Blau” object generated from *BSANet*.*rda*.

Ecology	Org.Niche	OrgMem	NicheMem	PredictedNicheMem	NicheExc	NicheOvr	MemExc	ExclusivePercent
New York	grp_conservative	2	2	2	1	1	0	50
New York	grp_liberal	2	3	2	2	1	0	66.67
San Francisco	grp_conservative	5	3	4	1	2	2	33.33
San Francisco	grp_liberal	4	4	4	2	2	0	50

A second output at the meso level–the “Focal Niche Summary” for each social entity–is given in Table 12. The column “OrgMem” lists how many actual members are in each social entity that construct its niche. The column “NicheMem” lists how many individuals are in each niche. The column “PredictedNichemem” lists the predicted number of individuals in the niche base, which is a product of the niche-by-niche matrix of competition coefficients in Table 11 and the column “OrgMem” in Table 12. The column “NichExc” lists how many individuals are exclusive to that niche and only to that niche. The column “NicheOvr” lists the number of individuals who overlap with other niches. The column “MemExc” lists how many individuals are actually members of the social entity but are not in its niche. The column “ExclusivePercent” lists the percentages of exclusives over all nichers. For example, the San Francisco conservative group has five actual members (i.e., Bryan, Wendy, Aude, Mona, and Bruce), three nichers (i.e., Bryan, Aude, and Mona), one exclusive (i.e., Bryan) with an exclusive percentage of 33.33%. It also has two niche insiders who are also located in the overlapping area with the niche of the San Francisco liberal group (i.e., Aude and Mona), and two actual members who are located outside of its niche (i.e., Wendy and Bruce).

A third output at the meso level–the bivariate “Correlation Matrix”–gives the correlation coefficients among the number of organizations and niches the node occupies and his or her values on the selected salient dimensions. As shown in Table 13, the correlation coefficient between the total number of organizations and niches is 0.73. Those between age and income, between age and educational level, and between income and education level are -0.03, 0.07, and -0.21, respectively. Since the three dimensions are not highly correlated with one another, each dimension provides non-redundant information and allows social differentiation along the other two dimensions. This suggests that the individuals’ locations on one dimension are not related to their locations on the other dimensions [1, 23]. Since, in this case, the network information is also available and loaded in memory, some network measures (e.g., out-degree, in-degree, and eigenvector centrality measures) are added to the correlation matrix. In this case, they are perfectly correlated.

**Table 13:** Correlation matrix for the "Blau" object generated from *BSANet*.*rda*.

Names	TotalOrgs	Niches	age	income	educ	outdegree	indegree	eigenvector
TotalOrgs	1	0.725542	-0.44798	0.313366	0.175665	0.313625	0.313625	0.313625
Niches	0.725542	1	-0.60862	0.148331	0.342997	0.272166	0.272166	0.272166
Age	-0.44798	-0.60862	1	-0.03402	0.066329	-0.23501	-0.23501	-0.23501
Income	0.313366	0.148331	-0.03402	1	-0.20987	-0.545	-0.545	-0.545
Educ	0.175665	0.342997	0.066329	-0.20987	1	0.035007	0.035007	0.035007
outdegree	0.313625	0.272166	-0.23501	-0.545	0.035007	1	1	1
Indegree	0.313625	0.272166	-0.23501	-0.545	0.035007	1	1	1
eigenvector	0.313625	0.272166	-0.23501	-0.545	0.035007	1	1	1

### Niche dynamics analysis

#### Cross-sectional analysis

Niche dynamics analysis is the fourth item under the “Analysis” menu. McPherson and Ranger-Moore [23] advanced a set of three measures–carrying capacity, membership rate, and intensity of exploitation. These can be calculated and plotted in Blaunet for cross-sectional exploration of how the niche of each social entity will move along the salient dimensions in Blau space. Like three-dimensional niche plot, Blaunet also allows its users to rotate the carrying capacity plot, the membership rate plot, and the intensity of exploitation plot. This rotation feature allows one to detect more details by selecting optimal values for horizontal or vertical angles.

**Fig 17:**
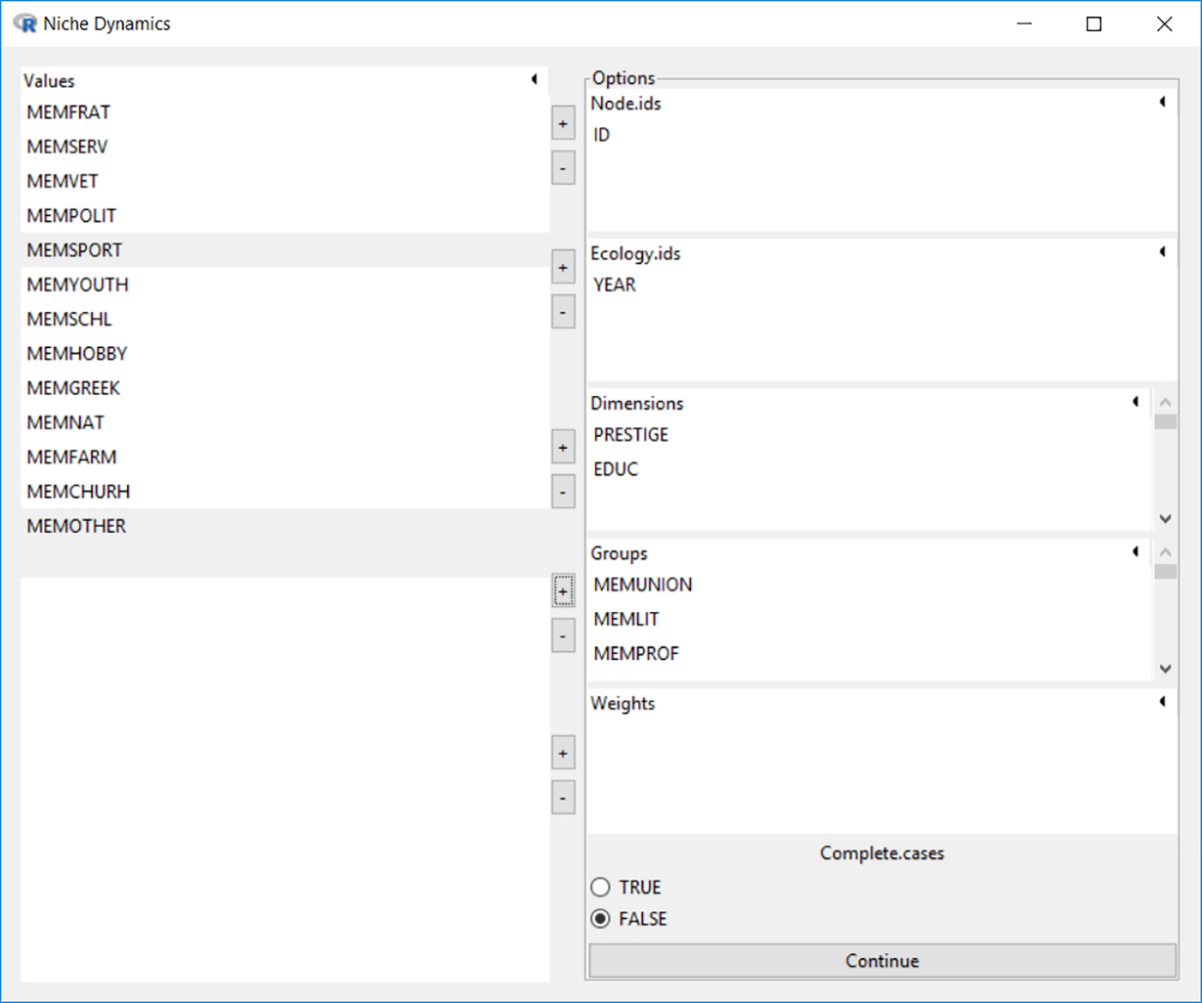
Interface for niche dynamics.

As shown in Fig 17 the example dataset *gss74_87*.*rda* is loaded in memory. The variable ID is selected as the node id, survey year (YEAR) as the ecology id, occupational prestige (PRESTIGE) and education level (EDUC) as the salient dimensions, and memberships in labor unions (MEMUNION), literary or art groups (MEMLIT), and professional groups (MEMPROF) as the group affiliation indicators.

**Fig 18:**
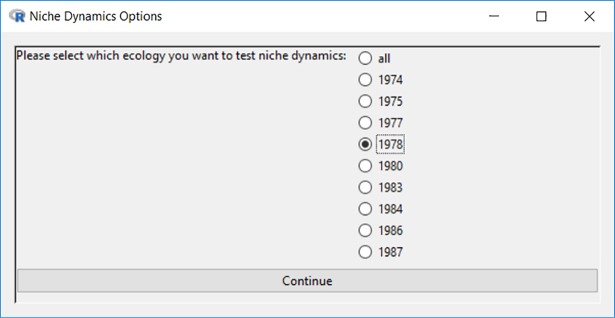
Interface for selection ecology.

After setting the deviation rage for each salient dimension, the survey year 1978 is chosen to illustrate the feature of cross-sectional niche dynamics analysis, as shown in Fig 18.

**Fig 19:**
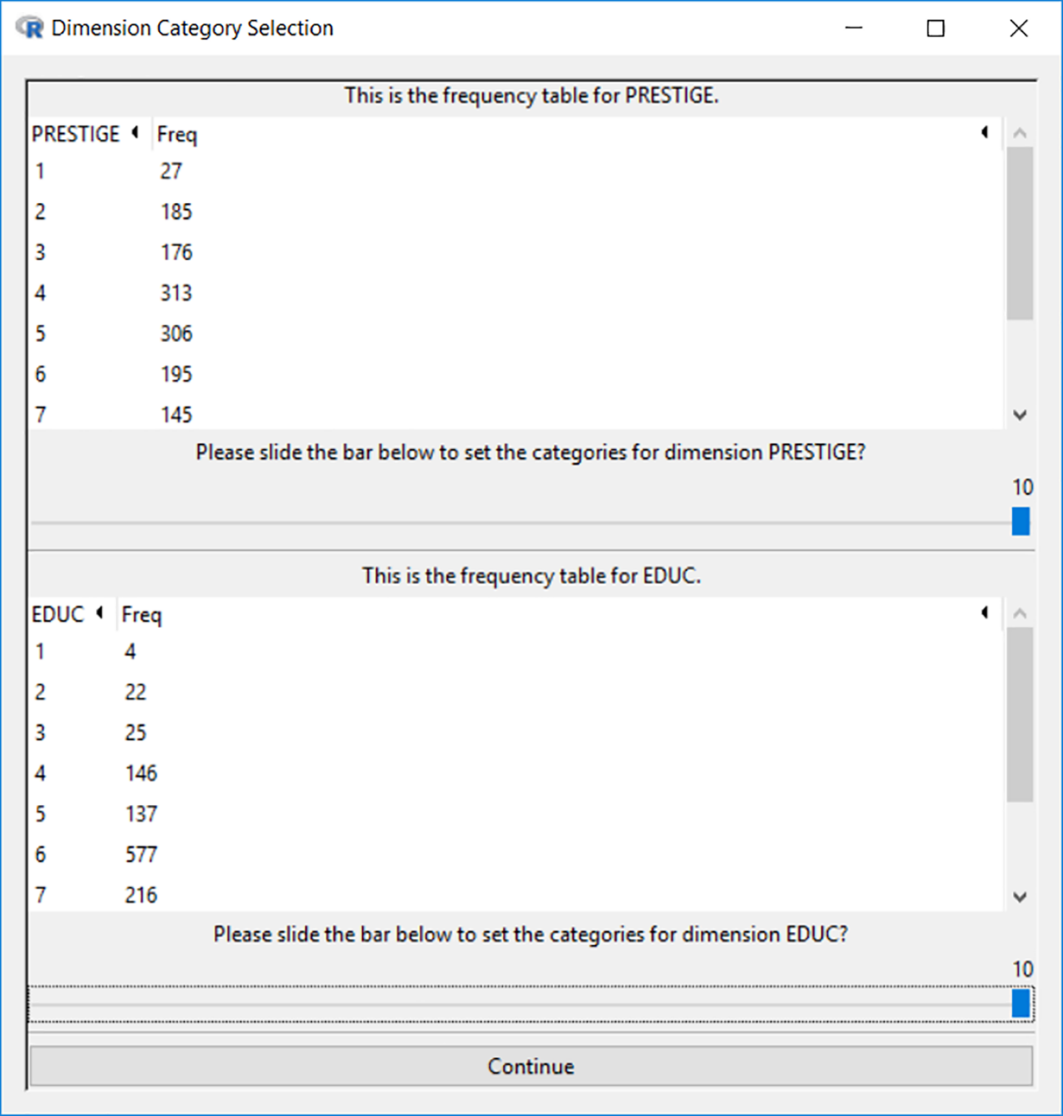
Interface for re-categorizing the 2 selected dimensions.

Blaunet allows its users to re-categorize the salient dimensions from continuous variables to categorical ones. In *gss74_87*.*rda* both occupational prestige and education level have been re-coded into 10 categories, so we only need to drag the handle to a maximum value of 10 for each salient dimension, as shown in Fig 19.

**Fig 20:**
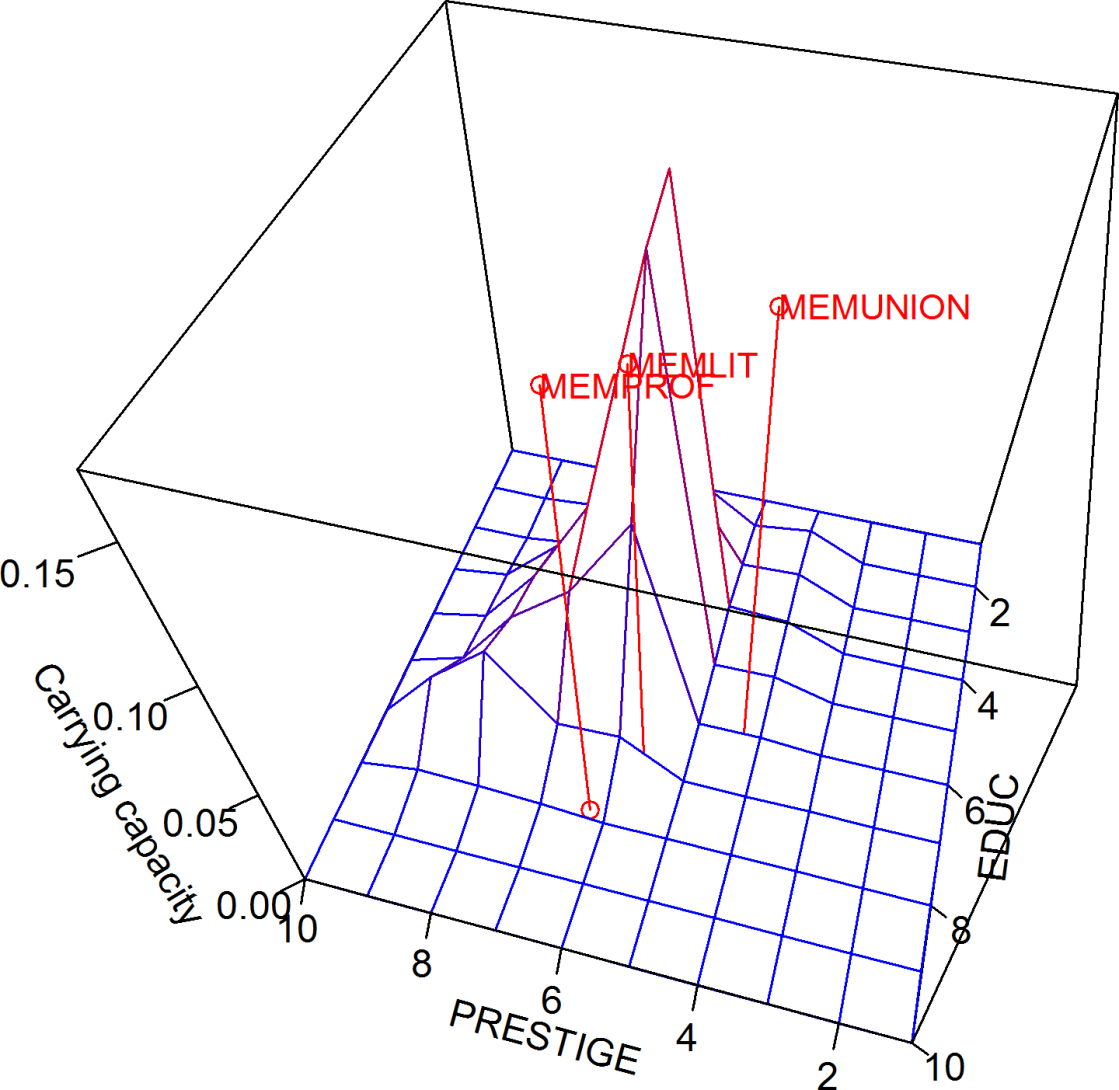
Carrying capacity in three-dimensional Blau space.

The carrying capacity indicates the distribution of potential members–or niche insiders–for selected social entities. As shown in Fig 20, both education level and occupational prestige are categorized into 10 equal intervals, resulting in 100 quadrants in the two-dimensional space. The proportions of niche insiders over the 100 quadrants are averaged across 3 selected social entities–labor unions, literary or art groups, and professional groups–and plotted along the vertical axis in the three-dimensional plot. The red color surrounds the peak where carrying capacity reaches its maximum value, while the blue color covers the flat areas where there are almost no potential members. Centered on the mean education level and mean occupational prestige of their actual members, all three types of organizations are located at areas where the carrying capacity is not very high.

**Fig 21:**
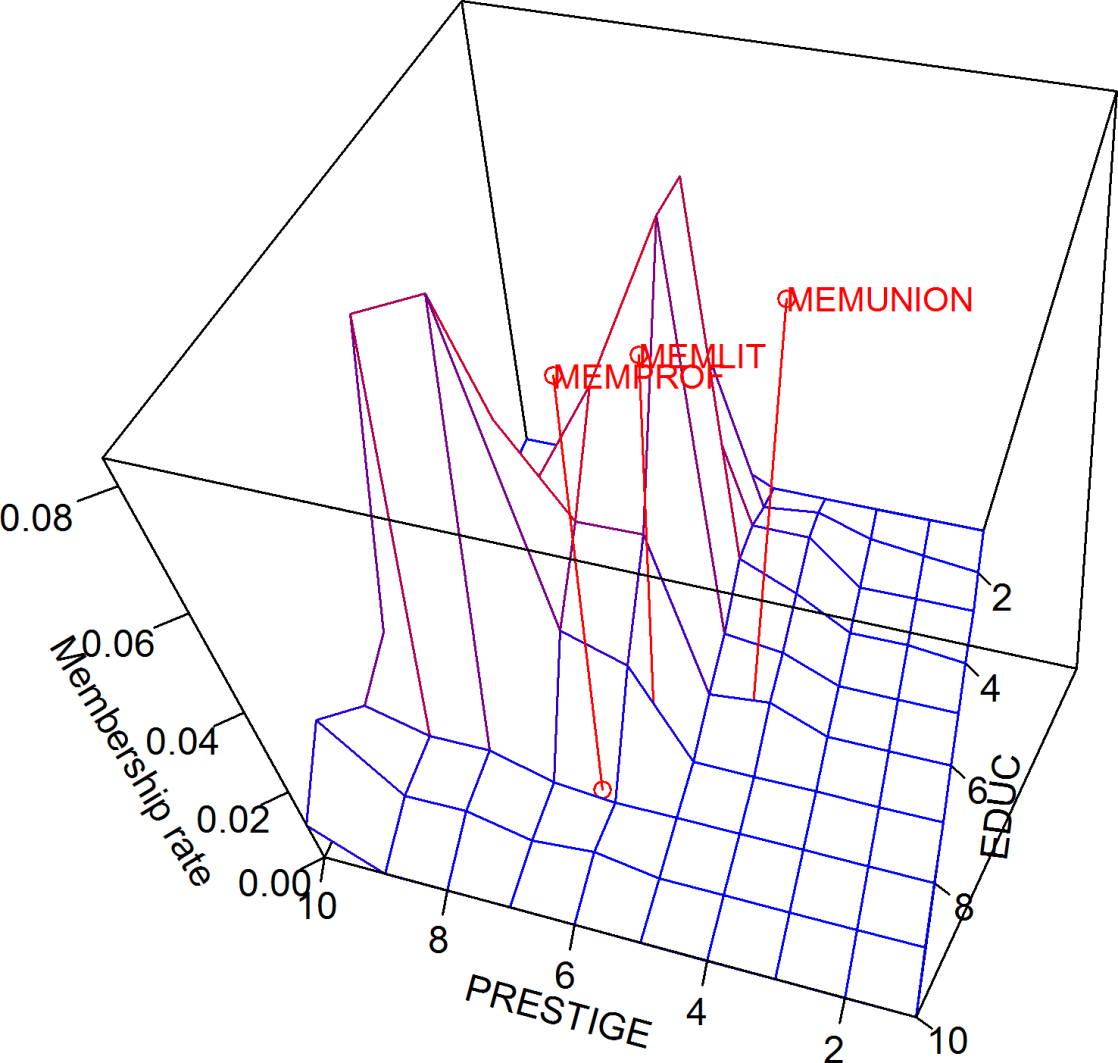
Membership rate in three-dimensional Blau space.

The only difference between the carrying capacity and the membership rate is that the latter refers to the distribution of actual members instead of potential members, over the 100 quadrants. As shown in Fig 21, all three groups are located at areas where the membership rate is not so high either.

**Fig 22:**
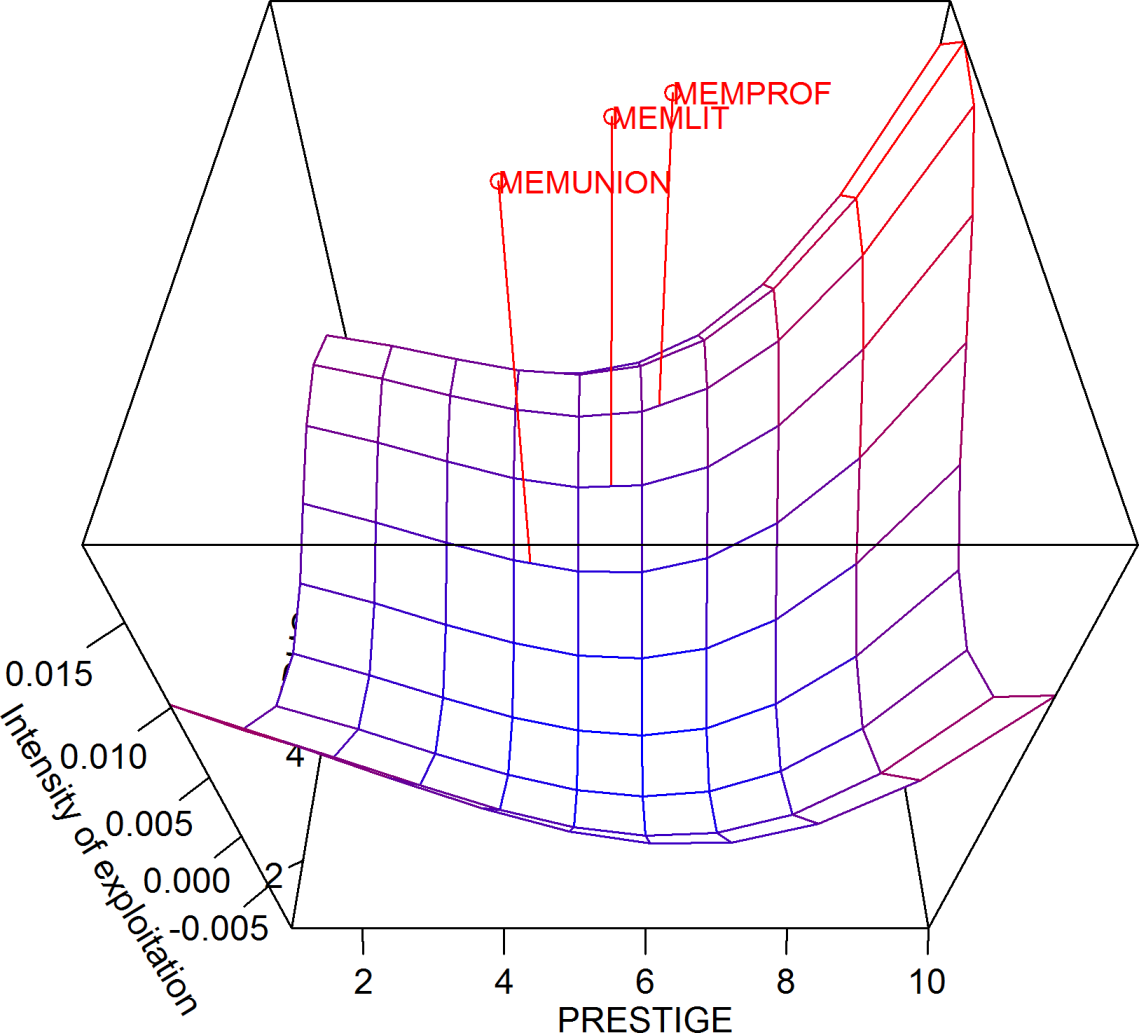
Intensity of exploitation in three-dimensional Blau space.

The intensity of exploitation plot shows the competitive dynamic that drives the gradient of high and low opportunities for social entities in Blau space. To estimate this measure, the arithmetic difference between the carrying capacity and the membership rate is calculated. Next, the surface constructed by the 100 quadrants is smoothed using a polynomial regression model. The polynomial regression equation in this case is *Y* = 0.02468601–0.01529415*PRESTIGE + 0.002871434*PRESTIGE^2^–0.0001603333*PRESTIGE^3^–0.001731003*EDUC– 0.0004662787*EDUC^2^ + 0.00006042156* *EDUC*^3^ + 0.0002174276*PRESTIGE*EDUC. These procedures are implemented in Blaunet automatically. As shown in Fig 22, social entities are expected to move away from the red peak areas where potential members are overexploited and towards the blue valley areas where potential members are underexploited [23]. In the example data, we can see that professional groups are located at an area with great opportunities to gain more members with lower levels of both occupational prestige and education, suggesting a niche movement in this direction. Literary or art groups would be expected to keep their niche position over the same level of occupational prestige but may move their niche position toward the area of Blau space with lower levels of education. Labor unions are expected to move their niches to areas with a relatively higher level of occupational prestige but a lower level of education.

#### Longitudinal analysis

McPherson and Ranger-Moore [23] have also used the predicted niche movement equations to model the niche dynamics longitudinally. When the example dataset *gss74_87*.*rda* is loaded in Blaunet, we have sociodemographic and group affiliation information collected along 2 dimensions–occupational prestige and education–for 16 types of organizations over 9 time points. We let Blaunet know that we plan to perform longitudinal analysis by selecting “all” in Fig 18, instead of a single year 1978. The dependent variable is the niche movement, indicated by the observed change in the means along each dimension for each type of organization across 8 = 9–1 time periods. Carrying capacity, membership rate, and intensity of exploitation introduced earlier in this subsection are now calculated for the first 8 time points. For each type of organization at each time point, 10 points are evenly selected on the right side between the mean (which is not included in the 10 points) and the upper bound of niche breadth (i.e., mean + 1.5 standard deviation by default). Then, another 10 points are evenly selected on the left side between the low bound of the niche breadth (i.e., mean– 1.5 standard deviation by default) and the mean (which is not included in the 10 points) and along each dimension. Since the mean itself is not included, for occupational prestige the quadrants for the 10 points on each side are [Mean_OCC_ ± (0.15, 0.30, 0.45, 0.60, 0.75, 0.90, 1.05, 1.20, 1.35, 1.50) *SD_OCC_, Mean_EDUC_]. For education the quadrants for the 10 points on each side are [Mean_OCC_, Mean_EDUC_ ± (0.15, 0.30, 0.45, 0.60, 0.75, 0.90, 1.05, 1.20, 1.35, 1.50) *SD_EDUC_]. Note that the deviance range (1.5 by default) is configurable in Blaunet. Next, the values of exploitation in each dimension are cumulated on each side by inserting the quadrants of the 10 points into the polynomial regression equation automatically generated by Blaunet. If the right cumulant is greater than the left cumulant, the organization should move its niche to the under-exploited area in the left direction. If the right cumulant is equal to the left cumulant, the organization is in an equilibrium status and no niche movement is anticipated. And if the right cumulant is less than the left cumulant, the organization should move its niche to the under-exploited area to the right. In this way the explanatory variables are defined as

$${Net\_opportunity}_{t,j,k}={Left\_cumulant}_{t,j,k}-{Right\_cumulant}_{t,j,k}$$ (5)

where *t* varies over the first 8 time points, *j* varies over the 16 organizational types, and *k* varies over the 2 dimensions–occupational prestige and education. Finally, 128 = (9–1) * 16 records with two explanatory variables and two dependent variables for each record are generated to estimate the predicted niche movement equations as

$${Nich\_movement}_{t,j,OCC}=0.03067+0.98923*{Net\_opportunity}_{t,j,OCC}$$ (6)

$${Nich\_movement}_{t,j,EDUC}=0.09113+2.50393*{Net\_opportunity}_{t,j,EDUC}$$ (7)

The coefficient of net opportunity for occupational prestige in Eq (6) is not significant at the 0.05 level, but that for education in Eq (7) is significant at the 0.05 level. The R-squared values for Eq (6) and Eq (7) are 0.001823 and 0.06988, respectively, which suggests that occupational prestige might not be a salient dimension to build Blau space, in this case. (Note that our predicted niche movement equations are not identical to those in McPherson and Ranger-Moore [23]. This may be because they were using a raw GSS dataset with 23,356 correspondents, the sample size of which is about 1.68 times as large as that of *gss74_87*.*rda* available from the ICPSR website or the NORC website.)

### Other features of Blaunet

Blaunet also has other features, including setting the working directory, browsing attribute and network data, plotting network graphs with attributes, generating various network statistics, saving high-resolution 2D and 3D plots, exporting Blau space data for future analysis, and more. Additional details are provided in the S1 File *Manual for the Blaunet Graphic User Interface Package*.

## Discussion

There are many features of Blaunet that are not possible to document here, but we hope that this paper, together with the S1 file supplemental manual, serves as a useful introduction to the capabilities of the package as well as the theoretical framework behind it. Of course, questions will inevitably arise that are not answered here or in the package documentation. For this reason, users can join the Blaunet Users Facebook Group at https://www.facebook.com/groups/425015561030239/ to communicate with the authors.

Although Blaunet provides powerful features for its users, it is far from finished and future developments are envisaged. Specifically, we intend to tackle several topics in our future work.

First, one of the major limitations with existing Blau space techniques is the handling of categorical variables (e.g., gender, race, marital status, etc.), which in the current version can be included in Blau bubble (proximity) analysis but not in niche analysis. We are working on a method enabling both continuous and categorical variables to be used in niche analysis.

Second, it is reasonable to suppose that salient dimensions may work differently in building the Blau space for different social entities, as well as on constructing the Blau bubble for each individual. For example, sport teams might have some minimal criteria for personal athletic ability, while the study group may not be concerned with this dimension. In the next version, we hope to more explicitly incorporate weights that adjust the niche and Blau bubble for the relative importance of different dimensions, permitting more accurate models.

Third, the underlying dynamic of this approach has been the competition for members between social entities. But a more realistic representation may include other relational dynamics besides competition, such as cooperation or symbiosis. For example, the Civil Rights Movement in the United States witnessed cooperation between various religious, political, and civic organizations and the building on each other’s membership in civil right movements. We are thus considering in incorporating multiple relationship dynamics among social entities in the next version.

## Supporting information

S1 File Manual for the Blaunet Graphic User Interface Package. (PDF)
