# Supplementary material for: Blaunet: An R-based graphical user interface package to analyze Blau space
Source: PLoS One. 2018 Oct 1;13(10):e0204990. doi: 10.1371/journal.pone.0204990 (PMC6166986; doi:10.1371/journal.pone.0204990)
Supplement: S1 File — (PDF) [file pone.0204990.s001.pdf]

Manual for the Blaunet Graphic User Interface Package

Table of Contents

1. Installation 1

2. Start Blaunet Graphic User Interface Package 2

3. Set Working Directory 3

4. Data Menu – Load and Unload Data 3

5. Browse Menu – Browse Data 5

6. Network Menu – Generate Network Statistics 5

7. Graph Menu – Graph Function 6

8. Analysis Menu – Analysis Function 6

9. Help Menu 17

10. Example Data 17

11. References 19

1. Installation

Users are recommended to have the most recent version of R installed. Thirteen dependent packages are installed along with the Blaunet graphic package, which may *take a longer time during the first time of installation*. These thirteen dependent packages include:

(1-4) 'gWidgets', 'gWidgetsRGtk2', 'RGtk2', and 'cairoDevice', which function to build the graphic interface;

(5-7) 'plot3D', 'plot3Drgl', and 'rgl', which function to make a 3D plot;

(8-11) 'network', 'sna', 'ergm', and ' statnet.common', which function to do network operations and analysis; and

(12-13) 'haven' and 'foreign', which function to import and export data file between R and other statistical software, such as SAS, SPSS, Stata.

1.1 Windows Installation

- Open R and install Blaunet in R via
  R> install.packages('Blaunet', repos="http://cran.r-project.org", dependencies=TRUE)

1.2 Linux Installation

Blaunet installation under Linux needs building the X11 and GTK+ environment.

- Hit the keyboard shortcut Ctrl+Alt+T to open 'Terminal'
- If the dependent library 'x11' is not installed, install it by typing:
  sudo apt-get install libglpk-dev
- If the dependent library 'gtk2.0' is not installed, install it by typing:
  sudo apt-get install libgtk2.0-dev
- If the dependent library 'rgl' is not installed, install it by typing:
  sudo apt-get build-dep r-cran-rgl
- Type 'R' in terminal and install Blaunet in R via
  R> install.packages('Blaunet', repos="http://cran.r-project.org", dependencies=TRUE)

1.3 OS X Installation

Blaunet installation under OSX requires the building of the X11 and GTK+ environment.

- Enter 'System Preferences', click 'Dock', and make sure 'Automatically hide and show the Dock' is checked.

- Hit the keyboard shortcut shift+command+u to open the 'Utilities' folder and then open 'Terminal'.
- Type 'xcode-select --install' in terminal to install Xcode command line tools, which is required for installing 'Mac Ports' package (Note: The whole 'Xcode' package from App Store is not necessary).
- Download the 'Mac Ports' package from https://www.macports.org/install.php and install it (Note: The installation may take up to 20 to 30 minutes).
- Download the 'XQuartz' package from http://www.xquartz.org/ and install it. Log out and log back for XQuartz to take effect.
- Download the 'RGtk2' package from https://cran.r-project.org/src/contrib/RGtk2_2.20.35.tar.gz
- Download the 'cairoDevice' package from https://cran.r-project.org/src/contrib/cairoDevice_2.25.tar.gz
- In terminal, type:
  export PATH=/opt/local/bin:/opt/local/sbin:$PATH
  sudo port selfupdate
  sudo port install pkgconfig
  export PATH=/opt/pkgconfig/bin:$PATH
  sudo port install gtk2 (Note: The installation may take up to 100 to 150 minutes)
  export PKG_CONFIG_PATH=/opt/local/lib/pkgconfig
  R CMD INSTALL RGtk2_2.20.35.tar
  R CMD INSTALL cairoDevice_2.25.tar
- Type 'R' in terminal
- Install 'BlauNet' in R via
  R> install.packages('Blaunet', repos="http://cran.r-project.org", dependencies=TRUE)

2. Start BlauNet Graphic User Interface Package

- In R, type:
  library(Blaunet)
  blaunetgui()

- As shown in Figure 1, the main interface of Blaunet includes five elements – the menu bar, the toolbar, the text frame showing current working directory, the 'Set Working Directory' bar, and the general information of Blaunet, from top to bottom.

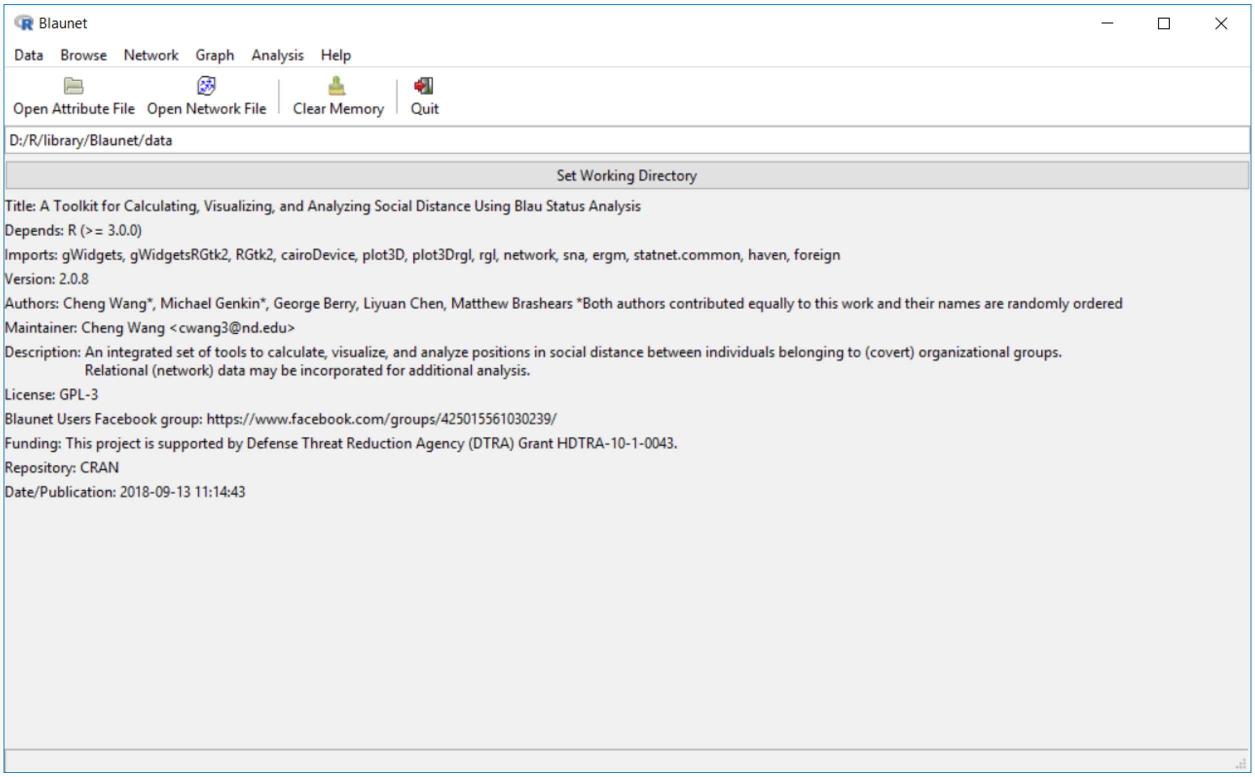

Figure 1: Main interface of Blaunet.

3. Set Working Directory

- The current working directory is shown in the text frame right below the toolbar.
- The working directory may be changed by clicking the 'Set Working Directory' bar from the main window on the Windows platform; on OS X or Linux platform, this can be done by typing the absolute path in the text frame and then clicking the 'Set Working Directory' bar.
- When the user selects to save any of the generated results, they will be saved into the working directory.

4. Data Menu – Load and Unload Data

Please be careful to ***NOT*** load the network data as attribute data or the attribute data as network data. It doesn't matter which one you load first.

4.1 Load the Attribute Data

- Multiple types of attribute data are supported, including R data (e.g., .rda & .Rdata), Stata data (e.g., .dta, version 14 or under), SPSS data (e.g., .sav), SAS transport data (e.g., .xpt), and other fixed width data (e.g., .csv, .data, and .txt).
- The first row of the attribute data should be variable names.
- The attribute data should be sorted by **a unique identification indicator for each node** (to link the attribute data with network data) as well as ecology if there are multiple sub-samples.
- The attributed data can be opened by clicking 'Data' → 'Open Attribute File' from the menu bar, or simply clicking the first icon from the toolbar.

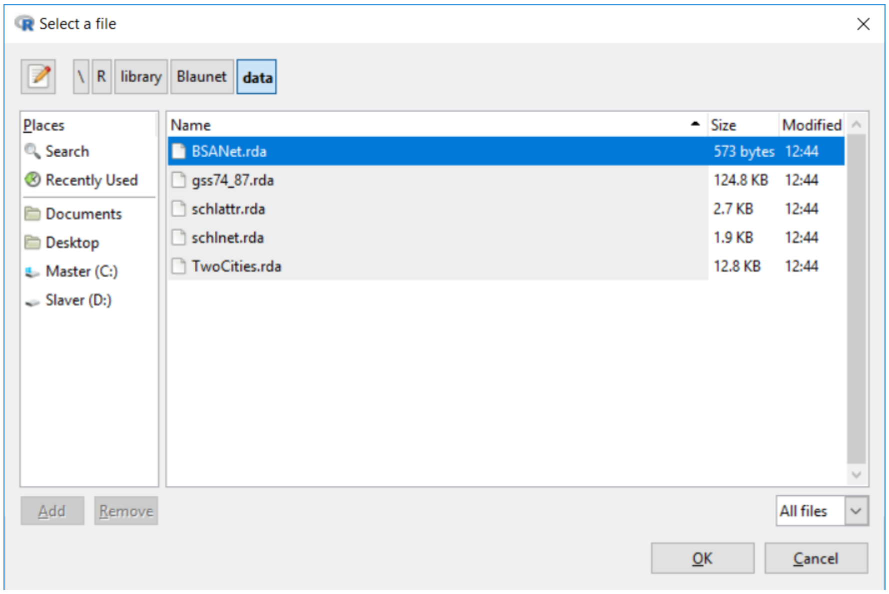

Figure 2: Interface for loading data.

4.2 Load the Network Data

- Multiple types of network data are supported, including R data (e.g., .rda & .Rdata), Pajek data (e.g., .paj), Stata data (e.g., .dta, version 14 or under), SPSS data (e.g., .sav), SAS transport data (e.g., .xpt), and other fixed width data (e.g., .csv, .data, .net, and .txt).
- The network data can be formatted as either an adjacency matrix or an edge list.
- The network data should ***NOT*** include any column names in the first row.

- The network data can be opened by clicking 'Data' → 'Open Network File' from the menu bar, or simply clicking the second icon from the toolbar.

4.3 Clear the Memory

- Before opening a new dataset the user should clear the memory, so that the data of the next project will not be corrupted up by the data of the previous project.
- This can be done by clicking 'Data' → 'Clear Memory' from the menu bar or the third icon from the toolbar.

4.4 Exit Package

- If the user wishes to exit the program, he or she may do so by clicking 'Data' → 'Quit' from the menu bar or the last icon from the toolbar.

5. Browse Menu – Browse Data

- Data can be browsed by clicking the 'Browse' button from the menu bar and selecting the correspondent data type one wishes to browse.

6. Network Menu – Generate Network Statistics

- Clicking 'Network' → 'Info' from the menu bar displays the summary information about the network data that has been loaded.
- Clicking 'Network' → 'Density' from the menu bar displays the network density.
- Clicking 'Network' → 'Centrality' from the menu bar displays the out-degree, in-degree, betweenness, closeness, and eigenvector centrality measures for each node. The results can also be saved into the working directory for later use.
- Clicking 'Network' → 'Dyad Census' from the menu bar displays the numbers of mutual, asymmetric, and null edges.
- Clicking 'Network' → 'Reciprocity Index' from the menu bar displays the proportion of mutual edges over all present ones.
- Clicking 'Network' → 'Triad Census' from the menu bar displays the numbers of 16 triadic isomorphism classes.
- Clicking 'Network' → 'Global Clustering Coefficient' from the menu bar displays the proportion of fully-connected triads over all 2-paths.

- Clicking 'Network' ⟶ 'Local Clustering Coefficient' from the menu bar displays the proportion of present edges among alters, over all possible ones for each node. The results can be saved into the working directory for later use.

7. Graph Menu – Graph Function

7.1 Plot the Network Graph

- After clicking 'Graph' ⟶ 'Network Graph' from the menu bar, a dialog box (as shown in Figure 3) is displayed where the user can choose whether vertex names/labels will appear in the graph, how to define the vertex color, sides (e.g., starts from a minimum of 3-sides or a triangle), and size, as well as what type of layout will be applied.

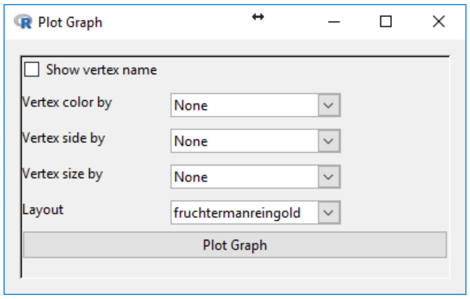

Figure 3: Options for plotting network graph.

- Click 'Plot Graph' to display the network graph. The network graph can be saved in different height, weight, resolution (in DPI), and file type into the working directory.

7.2 Histogram of Out-degree and In-degree Distribution

- Clicking 'Graph' ⟶ 'Histogram Out-degree' from the menu bar plots the out-degree distribution histogram. The histogram can be saved in different heights, weights, resolutions (in DPI), and file types into the working directory.
- Clicking 'Graph' ⟶ 'Histogram In-degree' from the menu bar plots the in-degree distribution histogram. The histogram can be saved in different height, weight, resolution (in DPI), and file type into the working directory.

8. Analysis Menu – Analysis Function

8.1 Salient Dimensions

- This feature provides a list of potential salient dimensions that may be used for

subsequent analysis. The potential salient dimensions are derived from network and membership variables. Therefore, the attribute data loaded should contain either membership and/or network variables.

- Clicking 'Analysis' → 'Salient Dimensions' from the menu bar opens a dialog box (as shown in Figure 4) and asks to specify the unique identification indicator for each node (as Node.ids), the ecology indicator if there are multiple sub-samples (as Ecology.ids), the set of dimensions, the group affiliation variables, and the alpha value. *Please note that it may take a longer time when the numbers of cases, dimensions, and groups is large.*

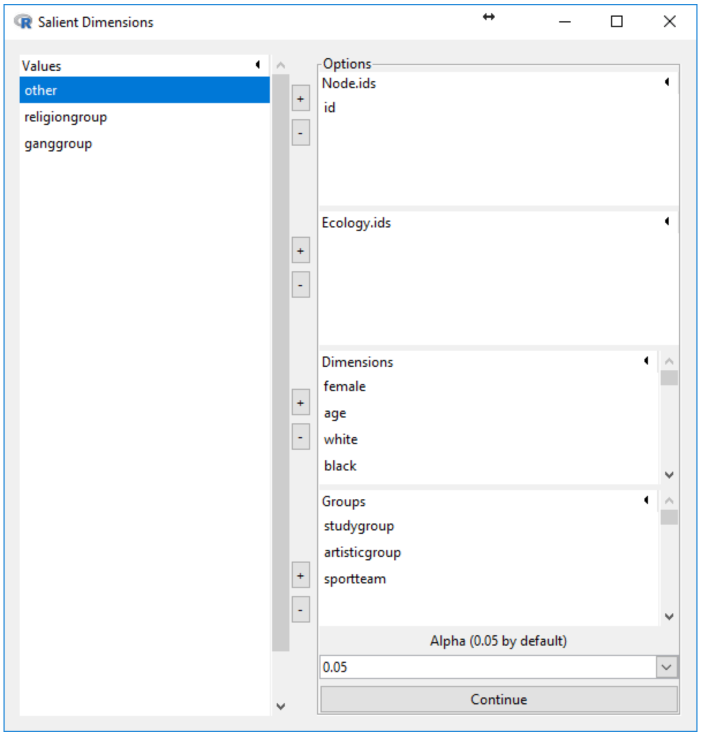

Figure 4: Interface for salient dimension identification.

- Click 'Continue', a new dialog box (as shown in Figure 5) opens up and asks to identify the categorical variables.

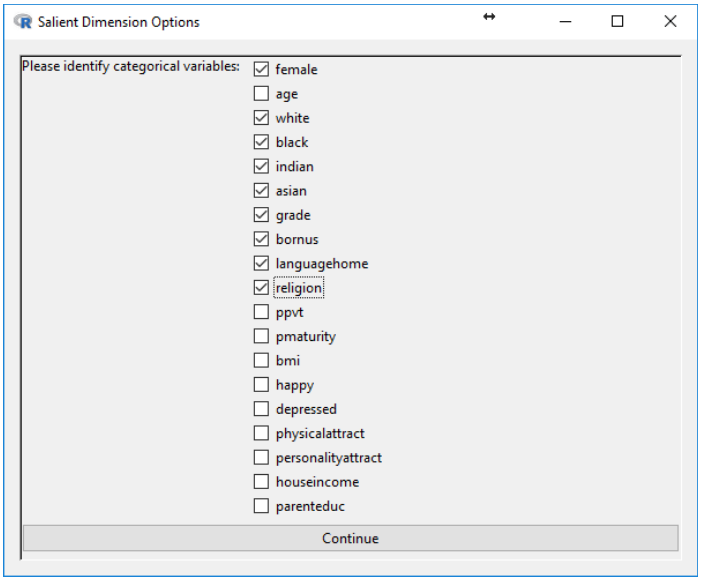

Figure 5: Identifying categorical variables among the set of dimensions.

- Click 'Continue' to get a list of potential salient dimensions (as shown in Figure 6). They may be used for conducting niche analysis (if continuous) or Blau bubble analysis (if the variables are either continuous or categorical).

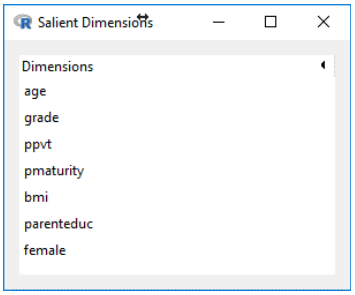

Figure 6: A list of potential salient dimensions

8.2 Niche Plot

- Clicking 'Analysis' → 'Niche plot' from the menu bar calls up a dialog box (as shown in Figure 7) which allows the user to input the unique identification indicator for each node (as Node.ids), the ecology indicator if there are multiple sub-samples (as Ecology.ids), whether the user wishes to display nodes and network ties in the plot, the 2 or 3 dimensions for the niche plot, the group affiliation variables, the sample weights if there are any, and whether or not only complete cases (with no missing data) are included.

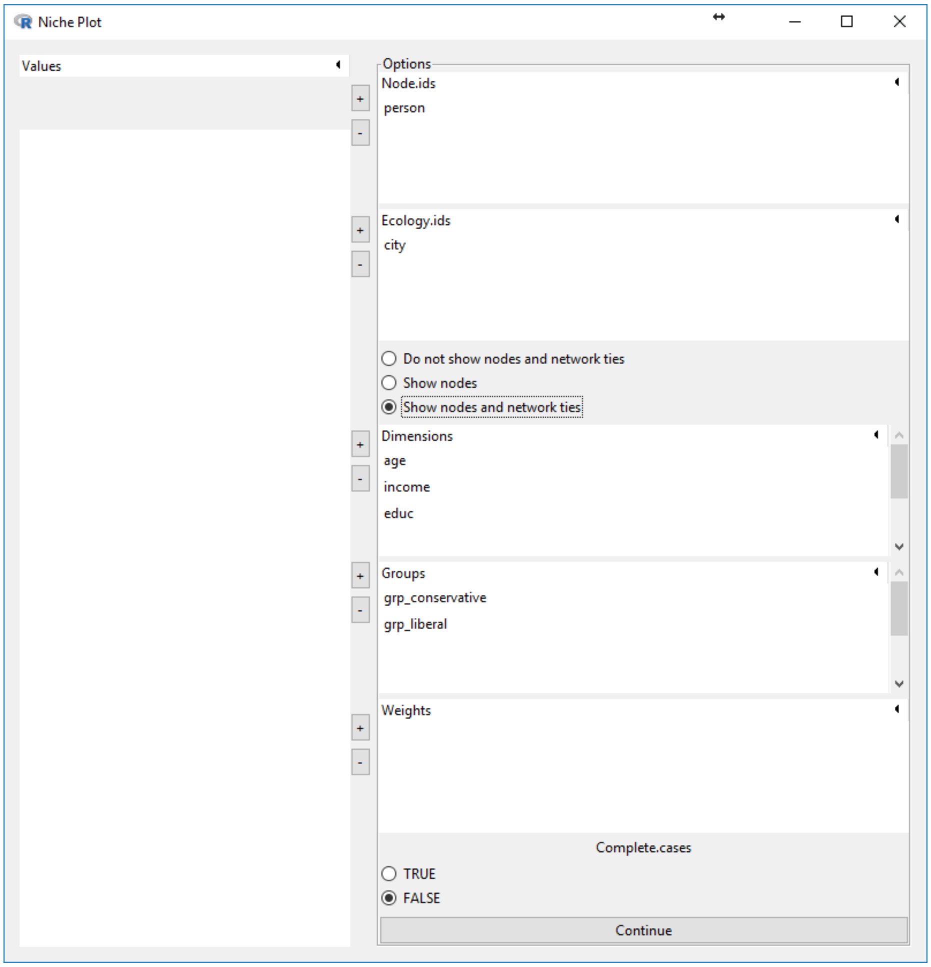

Figure 7: Interface for niche plot.

- After clicking the 'Continue' button, a new dialog box (as shown in Figure 8) asks to indicate which ecology to make niche plot if there are multiple sub-samples and specify the standard deviation around the mean (1.5 by default) for each dimension.

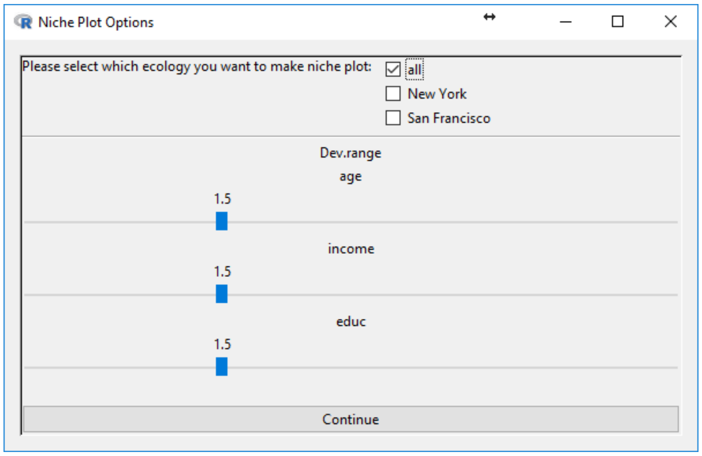

Figure 8: Options for niche plot.

- Clicking the 'Continue' button again, a rectangle (for 2 dimensions) or a cuboid (for 3 dimensions) is displayed for each group (and for each ecology if multiple sub-samples are specified) to indicate its niche along the selected dimensions. If the nodes or networks option is selected, all nodes or network ties will also appear overlaid on the niches. Selecting 3 dimensions allows the user to view two 3D plots side by side, one generated with 'plot3D' package which can be rotated by selecting values for horizontal or vertical angles and saved in different height, weight, resolution (in DPI), and file type into the working directory, and the other generated with 'plot3Drgl' package which can be freely rotated to any viewing points by dragging the mouse.

8.3 Niche Analysis

- Clicking 'Analysis' → 'Niche Analysis' from the menu bar, a dialog box (similar to the one shown in Figure 7) requests the unique identification indicator for each node (as Node.ids), the ecology indicator if there are multiple sub-samples (as Ecology.ids), whether or not network data is included, the dimensions, the groups, the sample weights if there are any, and whether or not only complete cases are included.
- Clicking the 'Continue' button, a new dialog box (similar to the one shown in Figure 8) asks the user to specify the standard deviation around the mean (1.5 by default) for each dimension.
- Clicking the 'Continue' button again, a small dialog box (as shown in Figure 9) is displayed with 6 buttons (a 7th button is displayed if network data is selected): *'Show Object*' browses the Blaunet object; *'Nodal Result*' shows for each node the number of groups he or she is affiliated with, the number of niches he or she is affiliated with, the niche list, three dummy variables indicating nodal status (outsider, insider/exclusive, and insider/manifolder), the personal affiliation state for each group and each niche, and the group/niche mixing state for each group; *'Niche Breadth Summary*' provides information about the lower bound and upper bound of each niche, along each dimension for each group (and for each ecology if multiple sub-samples are specified); *'Focal Niche Summary'* lists the number of individuals in each group, the number of individuals in its niche, the predicted number of individuals in the niche based on the competition coefficient matrix, the number of individuals exclusively in the niche, the number of

individuals who overlap with other niches, the number of individuals in each group but not in the group's niche, and the percentage of exclusives over all niche members; '*Niche by Niche Summary*' outputs a niche-by-niche matrix where diagonal elements contain the number of individuals exclusively in each group and the off diagonal elements correspond to the number of individuals overlapped in the niches, along with the competition coefficient matrix across niches (and ecologies) and its overall mean and standard deviation; and '*Correlation Matrix*' gives the correlation coefficients among the number of groups and the niches the node occupies along the selected dimensions.

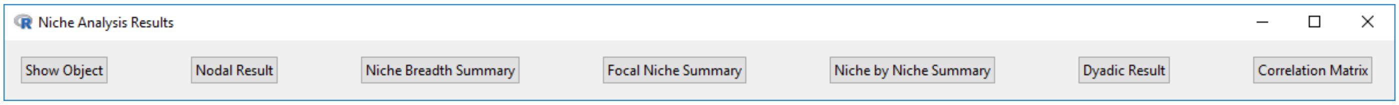

Figure 9: Interface for Blaunet object information.

- If the 'Network included' box is checked in the Niche Analysis dialog box, two extra columns appear in 'Nodal result': '*Spanner*' indicates whether each node spans to other niche(s) through his or her network tie(s) and '*NumSpannedTo*' indicates how many niche(s) he or she spans to. An additional 'Dyadic result' button shows up in the afore-mentioned small dialog box (as shown in Figure 9) which computes six dyadic measures for each present edge, including co-nicher, co-outsider, straddler, spanner, and Euclidean and Mahalanobis distance. Finally, out-degree, in-degree, betweenness, closeness, and eigenvector centrality measures are added to the 'Correlation Matrix' output.

8.4 Niche Dynamics

- Clicking 'Analysis' → ' Niche Dynamics' from the menu bar displays a dialog box (as shown in Figure 10), which requests the unique identification indicator for each node (as ecology.ids), the ecology indicator if there are multiple sub-samples (as ecology.ids), whether or not network is included, the dimensions, the groups, the sample weights if there are any, and whether or not only complete cases are included.

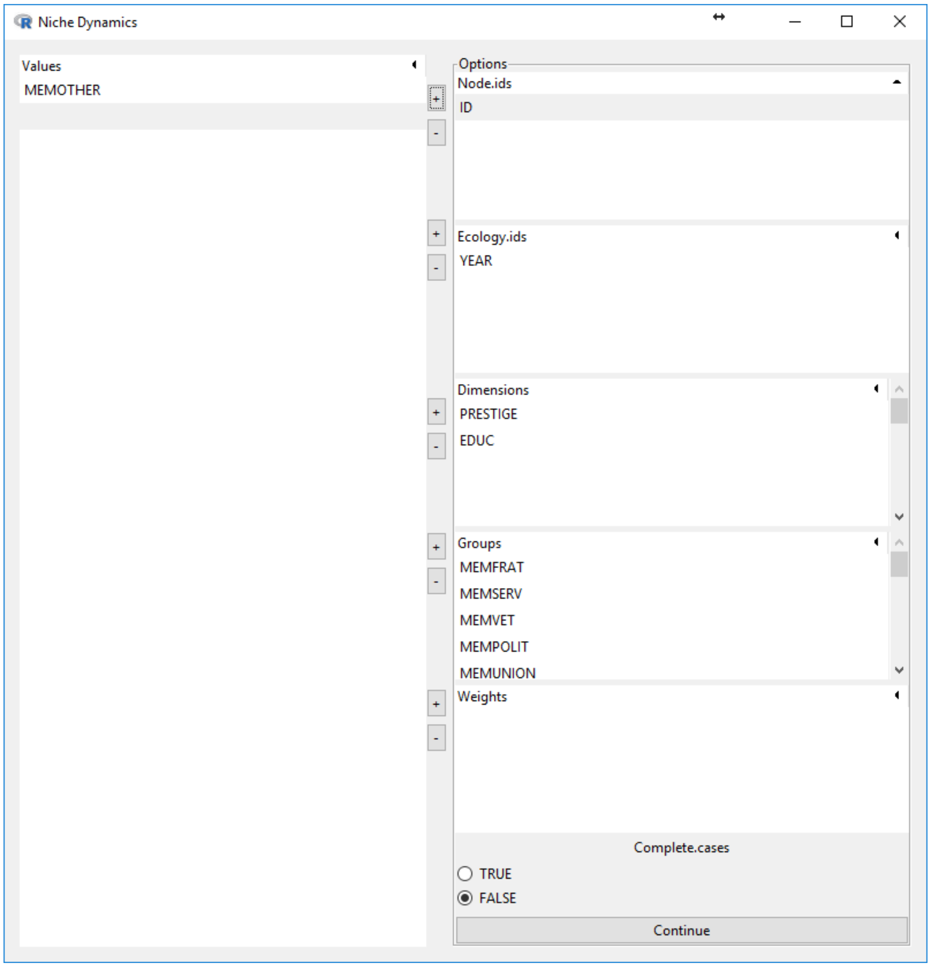

Figure 10: Interface for niche dynamics.

- Clicking the 'Continue' button, a new dialog box (similar to the one shown in Figure 8) asks to specify the standard deviation around the mean (1.5 by default) for each dimension.
- If there are multiple sub-samples/ecologies being specified (as ecology.ids) and the user wishes to see the predicted niche movement equations, he or she can click the 'Continue' button, select 'all' in the 'Niche Dynamics Option' dialog box (as shown in Figure 11), re-categorize the 2 selected dimensions in the 'Dimension Category Selection' dialog box (as shown in Figure 12), and find the results in the 'Predicted Niche Movements' dialog box (as shown in Figure 13). The results can be saved into the working directory for later use.

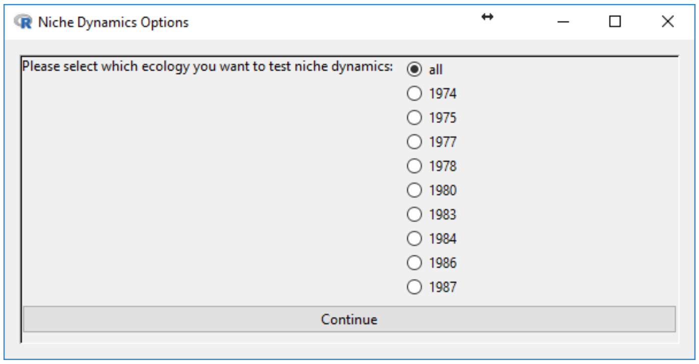

Figure 11: Interface for selection ecology.

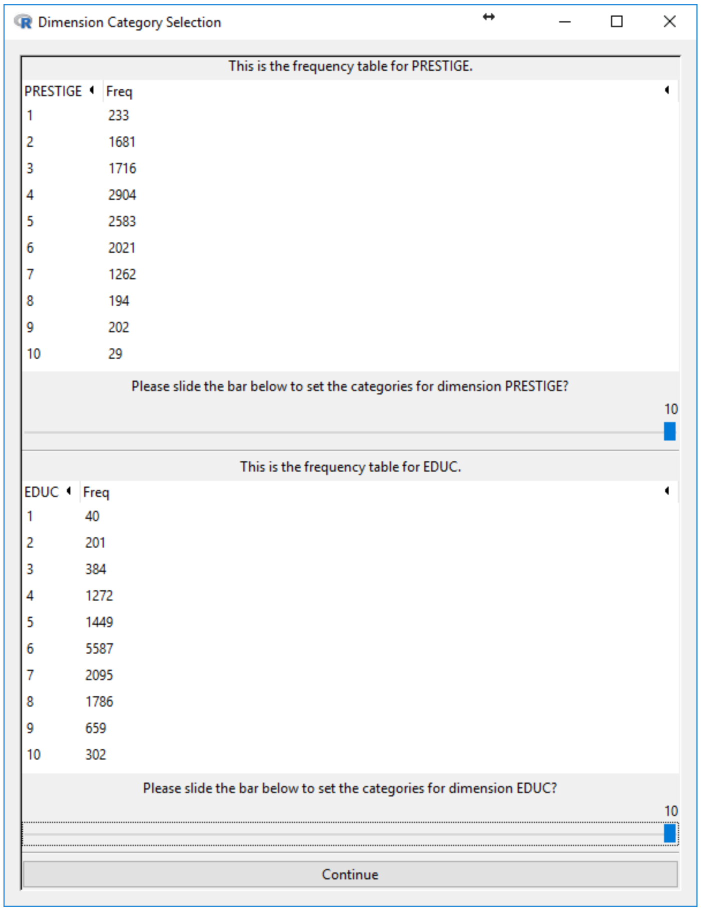

Figure 12: Interface for re-categorizing the 2 selected dimensions.

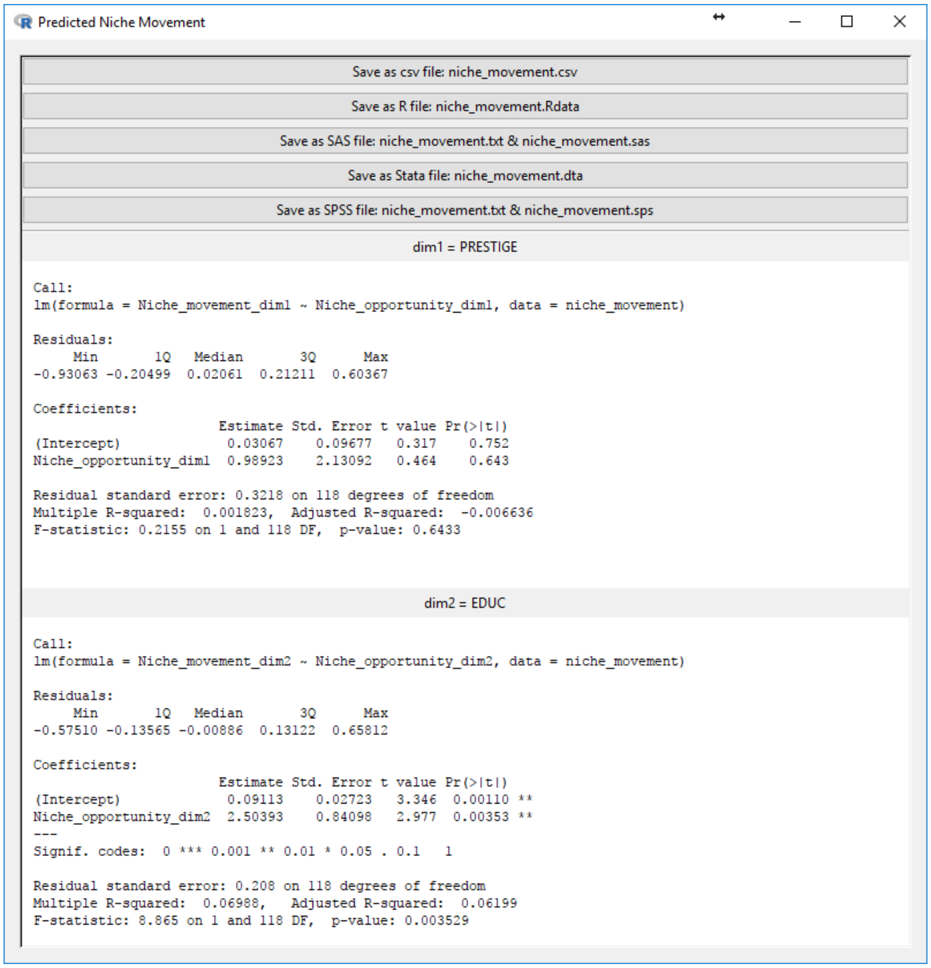

Figure 13: Predicted niche movement equations.

- If the user intentionally does not specify the ecology indicator (as ecology.ids), regardless of whether the data contains multiple sub-samples/ecologies, the user can still re-categorize the 2 selected dimensions in the afore-mentioned 'Dimension Category Selection' dialog box (as shown in Figure 12), and choose which plot he or she would like to see or just show the results in the 'Plot…' dialog box (as shown in Figure 14). The carrying capacity and membership rate plots show the distribution of niche members and actual members along the two re-categorized dimensions. The intensity of exploitation plot shows the polynomial regression model of the differences between the membership rate and carrying capacity along the two re-categorized dimensions, with coefficients from the polynomial regression model attached below the plot. The 'plot3D' package and 'plot3Drgl' package are used to generate two 3D plots side by side for each of the three plots mentioned above: one can be rotated by selecting values for horizontal or vertical angles and saved in different height, weight, resolution (in DPI), and file type, and the

other can be freely rotated along any viewing points by dragging the mouse. 'Show Table' outputs all the information about carrying capacity, membership rate, and intensity of exploitation. All the plots and results can be saved into the working directory for later use.

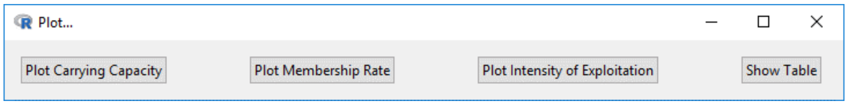

Figure 14: Predicted niche movement equations.

- If there are multiple sub-samples/ecologies being specified and the user wishes to make the plot for just one ecology, he or she can select the ecology in the afore-mentioned 'Niche Dynamics Option' dialog box (as shown in Figure 11), re-categorize the 2 selected dimensions in the afore-mentioned 'Dimension category selection' dialog box (as shown in Figure 12), and choose which plot he or she would like displayed or just save the results in the afore-mentioned 'Plot…' dialog box (as shown in Figure 14). All the plots and results can be saved into the working directory for later use.

8.5 Blau Bubbles (Blau Proximity Analysis)

- Clicking 'Analysis' → 'Blau Bubbles' from the menu bar displays a new dialog box (as shown in Figure 15) that requests the unique identification indicator for each node (as Node.ids), the ecology indicator if there are multiple sub-samples (as Ecology.ids), and the dimensions to generate Blau bubbles.

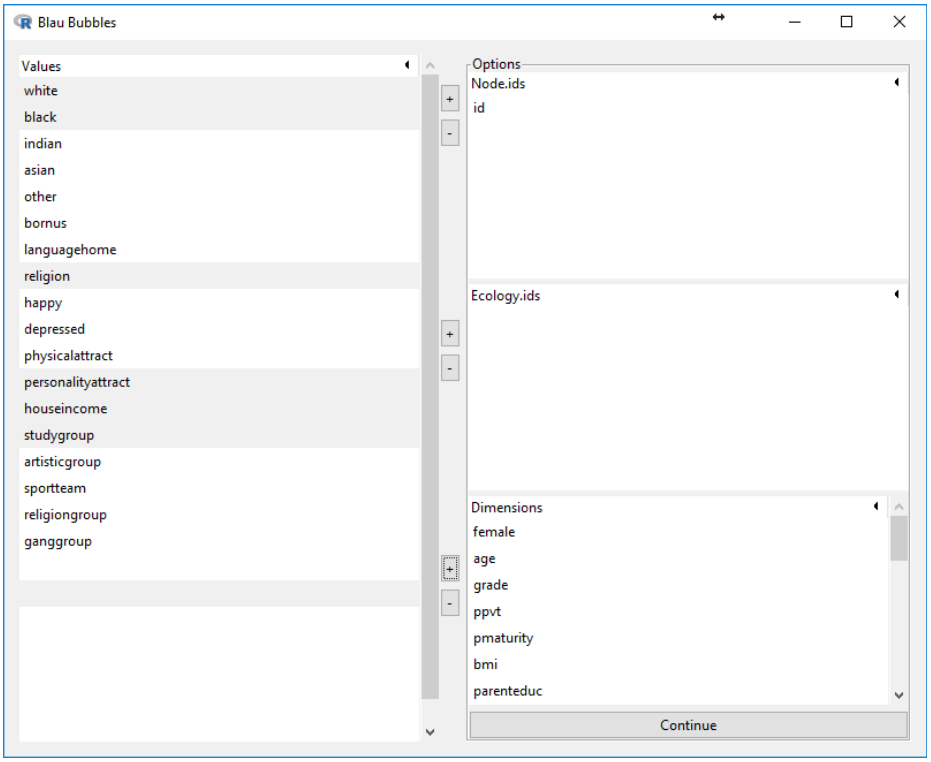

Figure 15: Interface for Blau bubble analysis.

- Clicking 'Continue' displays another dialog box (as shown in Figure 16) that asks the user to identify the categorical variables among selected dimensions and to define the radius (0.33 by default) of the Blau bubbles generated for each node.

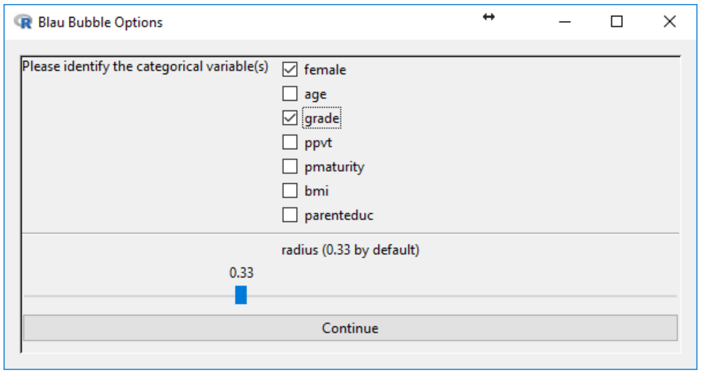

Figure 16: Blau bubble options.

- Click 'Continue' to see a small dialog box (as shown in Figure 17) with 4 buttons. 'Blau Distance Matrix' displays a square matrix indicating the Euclidean distance between any two nodes; 'Blau Bubble Matrix' displays a square matrix indicating whether or not any two nodes are in the same Blau bubble; 'Blau Bubble List' lists all pairs of nodes within the same Blau bubble and the distance between each pair of nodes (as dim.distance); and

'Nodal Bubble List' shows for each node how many others are in the Blau bubble as well as their IDs.

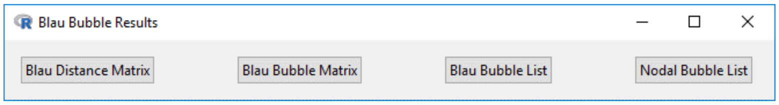

Figure 17: Blau bubble results.

- If network data are loaded in the program, 'Blau Bubble List' includes two additional measures indicating whether or not there is a tie between each pair of nodes and their geodesic distance in the network; and 'Nodal Bubble List' has four additional columns: the degree, alter list, number of coincidences that the alters are also in the Blau bubble as well as who they are.

9. Help Menu

9.1 About

- Clicking 'Help' → ' About' from the menu bar displays the Blaunet version information.

9.2 Graphic User Interface Package Manual

- Clicking 'Help' → 'Graphic Package Manual' from the menu bar displays the Manual for the Blaunet Graphic User Interface Package.

9.3 Command Line Manual

- Clicking 'Help' → 'Command Line Manual' from the menu bar displays the manual for the command line codes (mainly on niche analysis).

10. Example Data

10.1 BSANet.rda

- *BSANet.rda* is a small dataset containing 10 individuals in two non-overlapping locations (New York and San Francisco), created solely to illustrate the functions of the Blaunet package. It contains demographic information such as the individuals' age, education, and income; group affiliation information of memberships in a liberal or conservative organization (or both); and network information among the 10 individuals available in both adjacency matrix and edge list formats.

10.2 gss74_87.rda

- The *gss74_87.rda* dataset excerpted from the General Social Survey, 1972-2012, available from both the Interuniversity Consortium for Political and Social Research (ICPSR) website (http://www.icpsr.umich.edu/icpsrweb/NACJD/studies/34802) and the NORC website (http://gss.norc.org/get-the-data/). The demographic information includes the education level and occupational prestige scores of 13,865 respondents surveyed in 1974, 1975, 1977, 1978, 1980, 1983, 1984, 1986, and 1987; and the group affiliation information includes membership in 16 types of organizations. This is the dataset used in McPherson and Ranger-Moore (1991) though the number of cases used in the paper and in the example dataset is different.

10.3 schlattr.rda & schlnet.rda

- This data includes a set of 100 respondents is randomly drawn from the wave I public use data of National Longitudinal Study of Adolescent to Adult Health (Add Health), available from the Interuniversity Consortium for Political and Social Research (ICPSR) website (http://www.icpsr.umich.edu/icpsrweb/DSDR/studies/21600). The *schlattr.rda* file includes demographic information for 20 individual characteristics; and the group affiliation information for memberships in 5 kinds of organizations. The *schlnet.rda* file contains artificial network information that is simulated from an exponential family random graph (ERG) model.

10.4 TwoCities.rda

- The *TwoCities.rda* dataset is excerpted, with permission, from the Social Capital Benchmark Survey, which was collected by Professor Robert D. Putnam of the Saguaro Seminar: Civic Engagement in America, a project of the John F. Kennedy School of Government at Harvard University and numerous community foundations nationwide, and made available through the Roper Center for Public Opinion Research. The dataset contains 1,008 individuals from two cities in the United States: Bismarck, North Dakota, and Grand Rapids, Michigan. The demographic information includes 4 individual characteristics – age, income, education level, and work time; and the group affiliation

information includes membership in 18 voluntary organizations. There are also six other assorted variables.

More details on the key variable types (node.ids, ecology.ids, attributes, groups) as well as whether the dataset has a network component are summarized in Table 1 on the next page.

11. References

McPherson M. (1983). "Ecology of Affiliation". *American Sociological Review*, 48(4), 519–532.

McPherson M, Ranger-Moore J. (1991). "Evolution on a Dancing Landscape: Organizations and Networks in Dynamic Blau Space." *Social Forces*, 70(1), 19–42.

McPherson M. (2004). "A Blau Space Primer: Prolegomenon to an Ecology of Affiliation." *Industrial and Corporate Change*, 13(1), 263–280.

Table 1: Details of the four example datasets.

Dataset	Node.ids	Ecology.ids	Attributes		Groups	Network
			Continuous (Dimensions)	***Categorical***		
BSANet.rda	person	city	age, income, educ		grp_conservative, grp_liberal	Yes
gss74_87.rda	ID	YEAR	PRESTIGE, EDUC		MEMFRAT, MEMSERV, MEMVET, MEMPOLIT, MEMUNION, MEMSPORT, MEMYOUTH, MEMSCHL, MEMHOBBY, MEMGREEK, MEMNAT, MEMFARM, MEMLIT, MEMPROF, MEMCHURH, MEMOTHER	No
schlattr.rda & schlnet.rda	id		age, ppvt, pmaturity, bmi, happy, depressed, physicalattract, personalityattract, houseincome, parenteduc	female, white, black, indian, asian, other, grade, bornus, languagehome, religion	studygroup, artisticgroup, sportteam, religiongroup, ganggroup	Yes
TwoCities.rda	respID	samp	age, income, educ, worktime, trust, friends, divrsity	gender, race, ideo	grprel, grpsport, grpyouth, grppta, grpvet, grpnei, grpeld, grpsoc, grplab, grpprof, grpfrat, grpeth, grppol, grpart, grphob, grpself, grpwww, grpothr	No
